# Natural Sunlight IR‐Driven Highly Efficient Synthesis of Acetaldehyde From Bioethanol Over Cu/Fe_2_O_3_

**DOI:** 10.1002/anie.9513626

**Published:** 2026-08-05

**Authors:** Xiyi Li, Jiangting Zhao, Junjun Guo, Ze‐Kai Yu, Enqi Chen, Qiong Liu, Tieou Wang, Asterios Gavriilidis, Xue‐Qing Gong, Yang Lan, Junwang Tang

**Affiliations:** ^1^ Department of Chemical Engineering University College London London UK; ^2^ Industrial Catalysis Centre, Department of Chemical Engineering Tsinghua University Beijing China; ^3^ State Key Laboratory of Green Chemical Engineering and Industrial Catalysis Centre For Computational Chemistry and Research Institute of Industrial Catalysis School of Chemistry and Molecular Engineering East China University of Science and Technology Shanghai China; ^4^ Institute of Analysis Guangdong Academy of Sciences (China National Analytical Center, Guangzhou) Guangzhou China; ^5^ State Key Laboratory of Synergistic Chem‐Bio Synthesis School of Chemistry and Chemical Engineering Shanghai Jiao Tong University Shanghai China

**Keywords:** acetaldehyde, green hydrogen, IR‐driven catalysis, photon‐to‐phonon pathway, selective activation

## Abstract

Using renewable biomass to synthesize valuable chemicals can reduce fossil fuel dependence and achieve carbon neutrality. Here, for the first time an infrared light‐driven catalyst, Cu/Fe_2_O_3_, was designed to convert bioethanol to valuable acetaldehyde, accompanied by green hydrogen as a by‐product, under both indoor IR light and natural sunlight. It achieves an initial acetaldehyde yield of 237 mmol g^−1^ h^−1^ under indoor IR irradiation and 205 mmol g^−1^ h^−1^ under real sunlight, with exceptional selectivity (97.7%) and nearly stoichiometric H_2_ byproduct production. Notably, the turnover number and initial turnover frequency surpass those of IR‐driven systems by at least one order of magnitude and perform competitively with leading energy‐intensive UV–vis‐driven and thermocatalytic ethanol conversion processes operated up to 573 K. This high performance is attributed to: i) the construction of an efficient IR photons‐to‐phonons energy conversion channel within the ps timescale to drive localized thermocatalysis; and ii) the synergistic effect on the in situ formed of Cu/Fe_2_O_3_ interface, where Fe^3+^ sites promote dissociative ethanol adsorption, and Cu^0^ sites facilitates C─H bond cleavage.

## Graphical abstract

This work reports a Cu/Fe_2_O_3_ catalyst for the conversion of bioethanol into valuable acetaldehyde and green hydrogen under both infrared (IR) light and natural sunlight. This IR‐driven system sets a new performance benchmark, with TON and initial TOF enhanced by at least one order of magnitude, enabled by a constructed IR photon‐to‐phonon channel and the interfacial synergistic effect of Cu/Fe_2_O_3_.

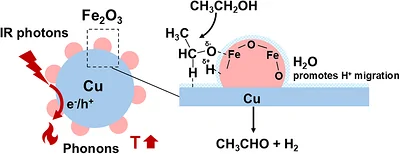

## 1 Introduction

Acetaldehyde is a crucial industrial building block, extensively utilized in organic synthesis for producing fine chemicals, perfumes, dyes, and pharmaceuticals [1, 2]. Its global consumption is approximately 1.4 million tons, with a market value exceeding USD 1.3 billion [1, 3]. The predominant commercial method for producing acetaldehyde is the Wacker–Hoechst process, which involves the direct oxidation of ethylene by O_2_ in an acidic catalytic solution of PdCl_2_ and CuCl_2_ [4, 5]. However, this process has several drawbacks: it uses minimal noble metals, generates chlorinated waste, suffers from corrosion issues, and relies on non‐renewable ethylene, which is inconsistent with sustainable development goals.

Biomass, especially bio‐based or bio‐derived materials from plants and foods, presents a substantial and renewable resource for exploring economically viable green synthesis [6]. Among them, bioethanol is one of the most common and renewable compounds on Earth, produced by biomass fermentation, which has the potential to yield 442 billion liters annually [7, 8]. The conversion of bioethanol to acetaldehyde offers economic potential, as the latter market price is approximately threefold higher than the former [9]. Thus, using bioethanol as a raw material for acetaldehyde production presents a highly incentivized alternative.

The conversion of bioethanol to acetaldehyde can proceed via two pathways: oxidative conversion (CH_3_CH_2_OH + 1/2O_2_ → CH_3_CHO + H_2_O) or anaerobic conversion (CH_3_CH_2_OH → CH_3_CHO + H_2_). The former pathway is thermodynamically favorable; however, the presence of O_2_ results in water as an oxidation byproduct and poses a risk for CO_2_ formation due to overoxidation and explosion if exceeding the flammability limit. Considerable interest exists in the latter reaction, which achieves 100% atomic utilization efficiency, with green hydrogen as the sole byproduct—a substance with significant market demand (e.g., 95 Mt in 2022) [10]. It should be noted that this reaction is thermodynamically uphill, requiring additional energy input (e.g., high temperature), which often leads to catalyst sintering or coke accumulation and increases the carbon footprint [11, 12, 13, 14].

Utilizing abundant UV and/or visible light as the sole energy source to refine biomass‐derived raw materials has recently emerged as a prevalent green technology [15, 16], which provides a chance to valorize bioethanol in a substantial and renewable way [6, 17, 18]. However, due to the lack of effective catalysts for selective bond activation and reaction pathway control while overcoming the sluggish kinetics of ethanol conversion, more attention has been devoted to hydrogen generation than to the value‐added oxidation products production [7, 19, 20, 21, 22, 23]. Environmentally unfriendly or costly bases/acids are often required to enhance ethanol conversion and facilitate the synthesis of carbon‐containing products [7, 19, 22, 23]. Notably, under neutral conditions, most reported systems exhibit only moderate production rates even with the addition of noble metals [17, 18, 24, 25, 26]. Another challenge in solar‐driven value‐added synthesis based on bioethanol is the incomplete utilization of the solar spectrum. All reported systems relied on UV/visible light, neglecting infrared light (IR, 700–2500 nm), which constitutes over 50% of the solar spectrum [6, 15]. This is because low‐energy IR photons are insufficient to excite charge carriers with adequate redox potentials for reactions [27]. Consequently, no sunlight/IR‐driven bioethanol valorization has been successfully demonstrated so far.

In this work, a range of metal oxides composed of abundant elements, known for their high IR photon absorbance or their potential ethanol activation were designed and prepared using the modified citric acid combustion method [28, 29, 30]. Upon rigorous screening, an infrared (IR)‐driven catalyst, Cu/Fe_2_O_3_, has been developed for the continuous acetaldehyde synthesis in a flow reactor without external heating, and has also been demonstrated under natural sunlight. The optimized Cu/Fe_2_O_3_ catalyst achieves high yields of acetaldehyde (237 mmol g^−1^ h^−1^) and hydrogen (232 mmol g^−1^ h^−1^) with 97.7% acetaldehyde selectivity. In particular, the turnover number (TON) and initial turnover frequency (TOF), based on either hydrogen or acetaldehyde production, outperform most photon‐driven systems and even conventional high‐temperature thermocatalytic processes. This exceptional performance arises from the construction of an IR photon‐to‐phonon conversion pathway and a synergistic effect at the in situ formed Cu/Fe_2_O_3_ interface.

## 2 Results and Discussion

### 2.1 IR‐driven Acetaldehyde Synthesis

A series of bimetallic metal oxides were synthesized and evaluated for their performance in the IR‐driven acetaldehyde synthesis based on bioethanol. As described in the Methods section, these catalysts were assessed using a flow reactor (Figure S1 and Note S1). In this setup, argon gas flowed through a liquid bubbler containing EtOH/H_2_O (molar ratio 1:1), thereby conveying the EtOH/H_2_O mixture vapor to the reactor for the IR‐driven reaction. Initially, a Xenon lamp equipped with a filter was used to provide the indoor IR photons only with wavelengths of 700–2500 nm (Figure S2), and the selected catalyst was then assessed under natural sunlight irradiation.

In the process of acetaldehyde synthesis from bioethanol, hydrogen (H_2_) is used as an indicator for catalyst screening (Figure 1a). Notably, the catalysts embedding Cu species (Cu/Fe_2_O_3_, Cu/MnO_x_, Cu/NiO_x_, Cu/CoO_x_) show elevated H_2_ yield rates among ten catalysts tested. This observation suggests that these samples are likely more effective in the ethanol conversion reaction and that Cu is likely the active catalytic center. In particular, the H_2_ yield rates over Cu/Fe_2_O_3_ and Cu/MnO_x_ are as high as approximately 53 mmol g^−1^ h^−1^. Following that, all carbon‐containing chemical products were analyzed to evaluate the performance of these four catalysts for acetaldehyde synthesis, as shown in Figure S3. A detailed examination of the product distribution on Cu/NiO_x_ and Cu/CoO_x_ reveals a higher yield rate of C_1_ products (CH_4_, CO), likely attributed to the unique capability of Ni and Co species to cleave the C─C bond of ethanol [28, 31, 32]. In contrast, Cu/Fe_2_O_3_ and Cu/MnO_x_ exhibit increased selectivity toward CH_3_CHO, reaching nearly 99%. To the best of our knowledge, this is the first report on IR photon‐driven acetaldehyde synthesis from ethanol up to date.

**FIGURE 1:**
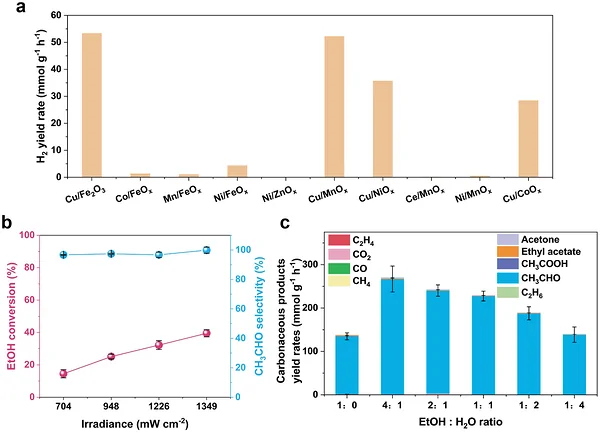
IR‐driven acetaldehyde synthesis. (a) Screening of bimetallic metal oxides based on H_2_ production rate during the IR‐driven acetaldehyde synthesis. (b) EtOH conversion and CH_3_CHO selectivity of the IR‐driven acetaldehyde synthesis over Cu/Fe_2_O_3_ under varying light intensities. (c) Effect of varying EtOH/H_2_O ratios on the carbon‐containing products yield rates. (Reaction conditions: (a) 30 mL min^−1^ Ar carrier gas, 60 mL EtOH/H_2_O (1:1) in the gas bubbler; (b) 60 mL min^−1^ Ar carrier gas, EtOH/H_2_O (molar ratio: 2:1), 0.01 mL min^−1^ liquid injection rate; (c) 120 mL min^−1^ Ar carrier gas, 0.02 mL min^−1^ liquid injection rate, 1226 mW cm^−2^ irradiance. All studies used 20 mg catalyst and 300 W Xe lamp with a 700 nm long‐pass filter (700 nm< *λ *<2500 nm). No external heating was supplied for all studies. The error bars were obtained from three independent runs.).

Moreover, Cu/Fe_2_O_3_, compared to Cu/MnO_x_, shows a CH_3_CHO/H_2_ ratio closer to 1 (Figure S4), indicating that the primary pathway on Cu/Fe_2_O_3_ is the ethanol conversion to acetaldehyde. The relatively higher CH_3_CHO yield compared to H_2_ on Cu/MnO_x_ likely attributes to the redox flexibility of Mn ions, which can modulate oxygen coordination and facilitate the lattice oxygen reduction in the reaction [33], so this catalyst performance is not durable. Following this initial screening, Cu/Fe_2_O_3_ has been identified as the most effective IR‐driven catalyst for subsequent studies, taking into account both the high yield rate and the high selectivity.

Then, the ratios of Cu to Fe were investigated (Figure S5). Either FeO_x_ or CuO_x_ individually exhibits a negligible H_2_ yield rate. However, the Cu/Fe_2_O_3_ catalyst with different Cu/Fe ratios results in an enhanced H_2_ yield rate. Specifically, the optimized molar ratio of Cu to Fe is 2:1, achieving a H_2_ yield rate of approximately 50 mmol g^−1^ h^−1^, indicative of a synergistic effect between Fe and Cu species in the reaction. (*Note*: This optimized ratio has been used for the subsequent investigation, denoted as Cu/Fe_2_O_3_.) The actual composition of the optimized sample was then determined by ICP‐OES (Table S1). The weight percentages of Cu and Fe are found to be 49.18% and 23.59%, respectively. These values closely align with the ratio of these elements in the precursor.

A control experiment was conducted, which clearly verifies that this process is driven by IR irradiation (see Figure S6 and Note S2). Subsequently, the reactants were introduced using a syringe pump rather than a simple gas bubbler to enable a more precise evaluation of catalyst activity in producing the valuable oxidation product (CH_3_CHO) and reduction product H_2_. This reaction system upgrade allows for precise control over the quantities of ethanol and H_2_O in the Ar flow, as shown in Figure S7.

The influence of IR irradiance was investigated as shown in Figure 1b and Figure S8. The yield rates of carbon‐containing products increase with rising irradiance (Figure S8a). For instance, the CH_3_CHO yield rate rises from ∼52 mmol g^−1^ h^−1^ under 704 mW cm^−2^ light irradiation to ∼167 mmol g^−1^ h^−1^ under 1349 mW cm^−2^ light irradiation. Additionally, the corresponding H_2_ production increases proportionally, resulting in an H_2_/CH_3_CHO ratio close to 1 (Figure S8b,c). It is worth highlighting that the selectivity for CH_3_CHO among all carbon products remains above 95%, despite the conversion of EtOH increasing to ∼40%, indicating effective suppression of overoxidation on the catalyst (Figure 1b). An irradiance of 1226 mW cm^−2^ was selected for the subsequent experiments to prevent overheating of the Xenon lamp.

Then, the GHSV was adjusted to manipulate the residence time of reactants over catalysts (Figure S9). A noteworthy more than 2.5‐fold increase in the yield rates of CH_3_CHO when the GHSV increases fourfold (Figure S9a). A similar trend can be observed for the byproduct H_2_ (Figure S9b). The enhancement in yield can be attributed to the more efficient mass transfer at higher GHSV. Furthermore, the selectivity to CH_3_CHO remains above 94%, and the H_2_/CH_3_CHO ratio is close to 1 (Figure S9c,d). Balancing the product yield rate and EtOH conversion, a GHSV of 360 000 mL g^−1^ h^−1^ was selected for the rest of the experiments.

Subsequently, the molar ratios of EtOH to H_2_O were optimized, as shown in Figure 1c and Figure S10. The presence of water can enhance the yield rates for this reaction, suggesting that water is a promoter for this reaction. Systems with lower water content (the EtOH/H_2_O ratio of 1:0 and 4:1) exhibit lower H_2_/CH_3_CHO ratios (0.47 and 0.71, respectively) compared to those containing more water (Figure S10a,b), highlighting the essential role of water in facilitating H_2_ evolution and maintaining relatively high H_2_/CH_3_CHO ratios, as discussed later. Notably, the yield of CH_3_CHO and H_2_ follows a volcano trend with increasing water content, with the EtOH/H_2_O ratio of 2:1 achieving the highest H_2_ yield with a near‐stoichiometric ratio (∼1) for CH_3_CHO production (Figure 1c and Figure S10a,b). Under this optimized EtOH/H_2_O ratio, the EtOH conversion reaches 27.8%, with a CH_3_CHO selectivity of 97.7% (Figure S10c). Excessive water may compete with ethanol for adsorption on the catalyst surface, resulting in fewer ethanol molecules available for the CH_3_CHO synthesis reaction. More importantly, CH_3_CHO selectivity is ranging from 95.4 to 98.3 across all ratios, suggesting that Cu/Fe_2_O_3_ is a promising catalyst to prevent overoxidation.

Therefore, the optimized reaction conditions are determined to be an irradiance of 1226 mW cm^−2^, a GHSV of 360 000 mL g^−1^ h^−1^, and an EtOH/H_2_O molar ratio of 2:1. The surface temperature is around 513 K, measured by placing a thermocouple at the center of the catalyst surface under IR irradiation. To provide a comprehensive view of the temperature distribution, thermal camera imaging was conducted (Figure S11). The temperature decreases from the center (red region, ca. 200–237°C) toward the edge (yellow region, ca. 160°C–190°C). Notably, the temperature at the center (237°C) closely matches the thermocouple measurement (240°C). Although a temperature gradient is evident, using the maximum temperature as a reference point is a more conservative approach when comparing with thermocatalysis. The stability of Cu/Fe_2_O_3_ under optimized conditions was evaluated (Figure S12 and Note S3). The production rates of both H_2_ and CH_3_CHO exhibit an initial decline during the first ∼70 h, followed by a slower decrease between 70 and 140 h, thereafter, the activity stabilizes at approximately 30 mmol g^−1^ h^−1^. As discussed later, the deactivation is attributed to the growth of Cu particle size. Importantly, the CH_3_CHO selectivity stabilizes at ∼98% despite occasional fluctuations, indicating that the primary reaction pathway is C_2_H_5_OH→CH_3_CHO+H_2_. Furthermore, the carbon balance remains stable, predominantly within 100 ± 10% (Figure S13). These results also suggest that catalyst deactivation is not caused by coke deposition. Although the activity ultimately stabilizes at ∼30 mmol g^−1^ h^−1^, lower than the initial value, it still exceeds all initial production rates reported for IR‐driven catalysts, whether compared by production rate (mmol g^−1^_cat_ h^−1^) or by TOF (mmol g^−1^_M_ h^−1^), as shown in Table S2. Long‐term stability data are generally scarce and challenging for photon‐driven catalysts, and only a few examples exceed 50 h of testing [17, 26, 34]. Further work is ongoing to develop strategies to mitigate sintering and enhance structural robustness.

To evaluate the practical application potential of this catalyst, outdoor experiments utilizing real sunlight were conducted during sunny periods on September 12, 2024, and September 19, 2024, as shown in Figure 2a,b. The products, CH_3_CHO and H_2_, were collected and analyzed hourly, as weather condition varies during the test period. The yield of products changes proportionally with the solar irradiance (a Fresnel lens was used to concentrate sunlight). Furthermore, the ratio of CH_3_CHO/H_2_ remain relatively close to 1 despite some fluctuations in seven runs. Notably, a yield as high as 4.1 mmol of CH_3_CHO has been measured during one sunny hour (9:30–10:30, September 19, 2024), which corresponds to 205 mmol g^−1^ h^−1^ CH_3_CHO on average, comparable to the optimized yield rate obtained indoors. This results in a solar‐to‐chemical conversion efficiency of 0.14%. This stable selectivity and high performance demonstrate the feasibility of directly using natural sunlight to continuously produce valuable acetaldehyde along with green hydrogen byproduct from bioethanol.

**FIGURE 2:**
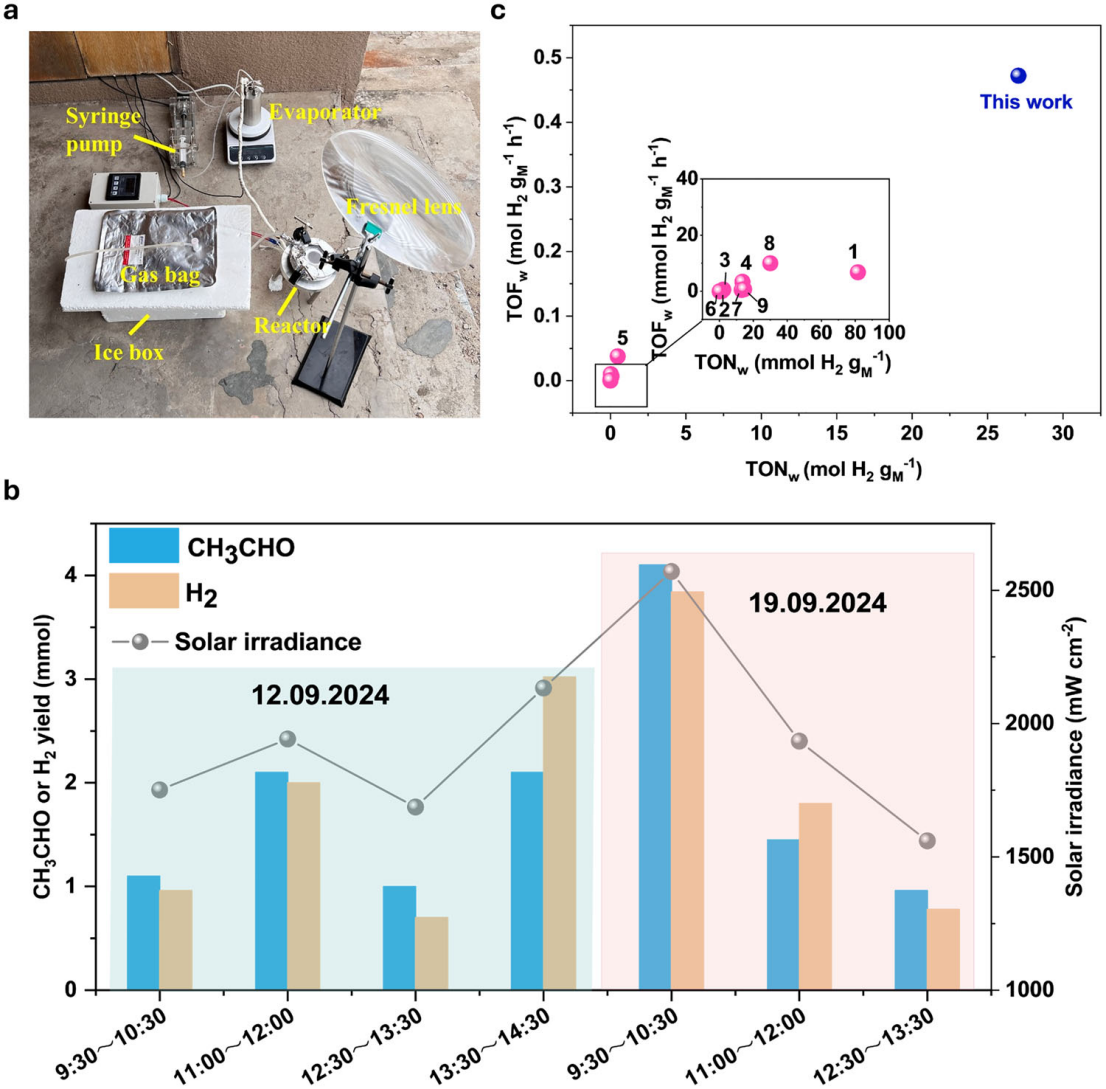
Activity under natural sun irradiation and representative results reported. (a) The reaction system for natural solar‐driven acetaldehyde synthesis reaction. (b) The outdoor experiment on acetaldehyde synthesis using real solar light over Cu/Fe_2_O_3_ (the reaction condition is detailed in the Method section). (c) TON_w_ and initial TOF_w_ of Cu/Fe_2_O_3_ and representative IR‐driven catalysts for H_2_ production (1: Black P NS/Pt/TiO_2_ [35]; 2: Au cube/cage‐TiO_2_‐Pd Cube [36]; 3: Pt/CuS/CdS [34]; 4: Black P/Pt/RGO [37]; 5: WS_2_/black phosphorous [38]; 6: TiO_2‐x_ [39]; 7: Black P/g‐C_3_N_4_ [40]; 8: Black P/Au/La_2_Ti_2_O_7_ [41]; 9: β‐NaYF_4_:Yb^3+^, Tm^3+^@Ag_3_PO_4_@Black P [42]). TON_w_ and initial TOF_w_ were calculated based on the weight of the active component for H_2_ production because surface active sites are generally not reported. Detailed reaction conditions can be found in Table S2.

As shown in Table S2, our study represents the first systematic investigation of both H_2_ and valuable carbon‐containing products generated under IR irradiation. To enable a comprehensive assessment of the performance, the turnover number (TON) and initial turnover frequency (TOF) were determined (Figure 2c; detailed definition and calculation procedures are provided in the Methods section of the Supporting Information). Using H_2_ as the indicator, our catalyst sets a new record in initial TOF_w_, surpassing all reported IR‐driven systems using EDTA or MeOH, and so forth, as the proton source by at least one order of magnitude. TON_w_, which captures both the activity and durability, further highlights the excellent performance of our catalyst, which exceeds most reported systems by two orders of magnitude. Even when normalized to irradiance, our work still leads by at least one order of magnitude. These observations indicate that long‐term durability remains a major challenge for IR‐driven catalysis, and our catalyst maintains the top performance among known IR‐driven systems. This exceptional performance of our catalyst even rivals the majority of the energetic UV–visible photon‐driven ethanol conversion systems as discussed in Figure S14 and Note S4.

Given the IR‐driven catalytic nature of this catalyst, comparison with thermocatalytic ethanol dehydrogenation systems is also essential (Figure S15 and Note S5). Most thermocatalysts exhibit initial TOF_a_ below 60 h^−1^, even at elevated temperature (up to 573 K). In contrast, this work achieves 370 h^−1^ under IR irradiation without external heating, with selectivity also among the top levels reported (Table S3). A control experiment was conducted to compare the energy efficiency of thermal heating and IR irradiation under similar conditions (Figures S16, S17 and Note S6), further showing the strong potential of solar IR light as an energy‐efficiency driving force.

### 2.2 Catalyst Characterizations

The dynamic changes of the active center under the realistic reaction conditions, particularly the chemical state of Cu species, often require a combination of in situ techniques to achieve a comprehensive understanding [43, 44, 45]. In situ XPS was performed first under the optimized reaction conditions, as shown in Figure 3a–c. Prior to the reaction, the peaks attributed to Cu 2*p*_3/2_ and Cu 2*p*_1/2_ at around 933.9 and 953.9 eV, along with the satellite peak, indicate the existence of Cu^2+^ (Figure 3a) [46, 47]. As the reaction progresses to 3 and 5 min, the intensity of these Cu^2+^ peaks gradually diminishes, while two new peaks emerge at around 932.5 eV (Cu 2*p*_3/2_) and 952.3 eV (Cu 2*p*_1/2_), which can be attributed to either Cu^+^ or metallic Cu(0) [46]. After 10 min of reaction, only the peak associated with Cu^+^ or metallic Cu(0) remain, suggesting a complete reduction of the Cu species. To further elucidate the chemical state of the Cu species, Cu LMM Auger spectra were analyzed (Figure 3b). With increasing the reaction time, the shape and position of the Cu Auger peak change, shifting from 917.6 to 916.7 eV and eventually 918.8 eV, corresponding to Cu^2+^, Cu^+^, and Cu(0), respectively [46, 48]. These results clearly indicate a rapid and progressive reduction of the Cu species in the catalyst over a short preactivation process under reaction conditions. On the other hand, the Fe 2*p* spectra exhibit two distinct peaks at 711.0 (Fe 2*p*_3/2_) and 724.5 eV (Fe 2*p*_1/2_), along with a satellite peak at approximately 719.0 eV (Figure 3c), all are characteristic of Fe^3+^ [49]. These peaks remain unchanged throughout the reaction period, with no observable shifts or the emergence of new features, indicating that the chemical state of Fe remains as Fe^3+^ under the reaction conditions.

**FIGURE 3:**
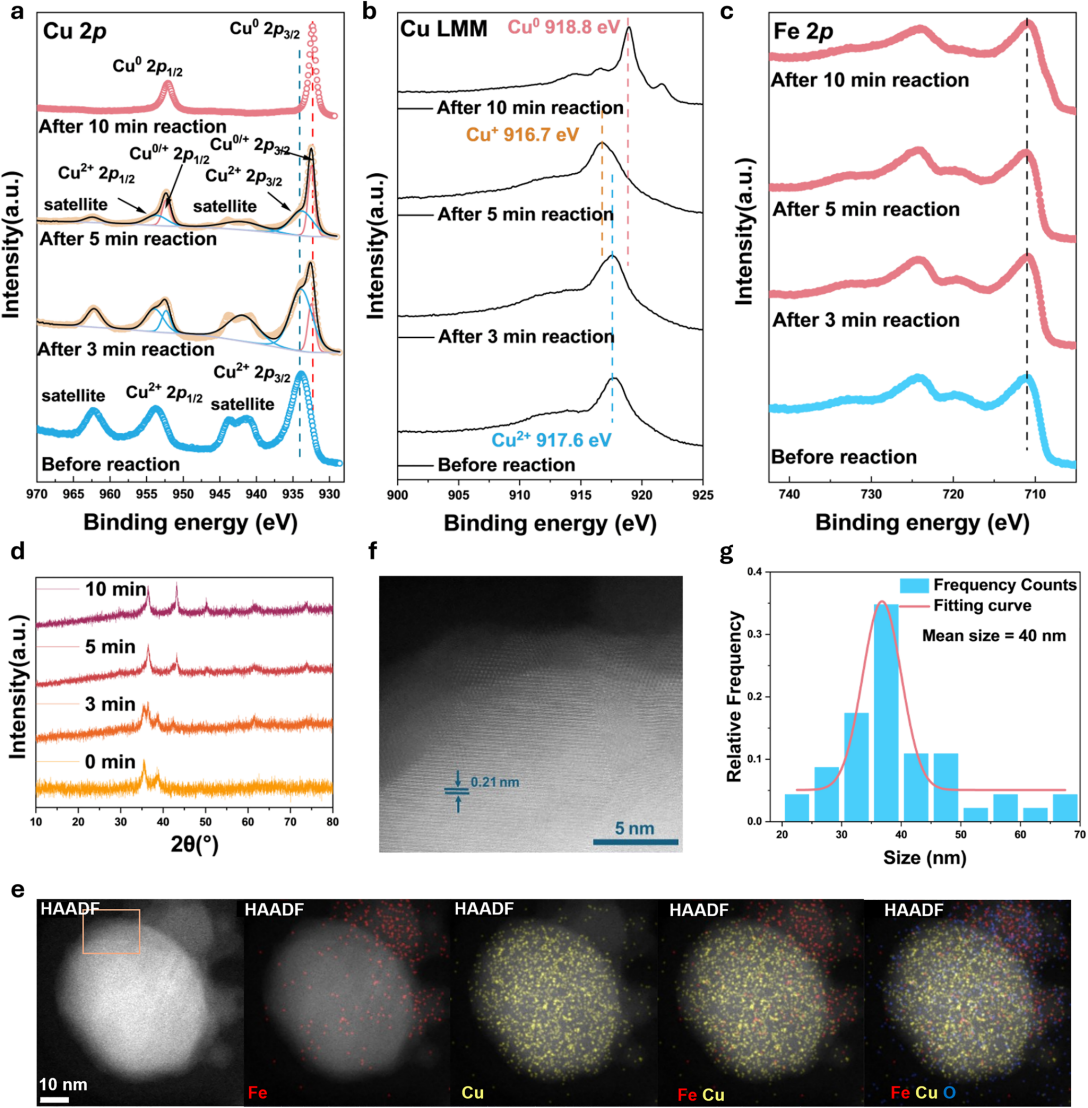
Structure characterization of Cu/Fe_2_O_3_. (a–c) The in situ XPS spectra of Cu 2*p*, Cu LMM and Fe 2*p* on Cu/Fe_2_O_3_ under the optimized reaction conditions. (d) The in situ XRD patterns of Cu/Fe_2_O_3_ under the optimized reaction conditions. (e) The HAADF‐STEM image and EDX element mapping of one representative large nanoparticle. (f) Zoom‐in of Figure 3e for the HR‐TEM analysis (the region highlighted by the orange rectangle in Figure 3e). (g) The particle size distribution of Cu nanoparticles in Figure S19a (*Note*: The fitting curve was generated by the Gauss model).

In situ XRD was also performed to further investigate the restructuring process (Figure 3d). Prior to the reaction, two main peaks were observed at approximately 35.5° and 38.6°. The peak at 35.5° can be attributed to combination of CuO(002) and Fe_2_O_3_(311), while the peak at 38.6° corresponds to CuO(111) [50, 51].After 3 min of reaction, the 35.5° peak begins to split with the appearance of a shoulder peak near 36.4°, which is likely associated with the formation of Cu_2_O with (111) facets [50]. Upon extending the reaction time to 5 min, a new peak emerges at ∼43.2°, which is characteristic of the (111) plane of metallic Cu [50, 52]. The intensity of this peak increases significantly with longer reaction time, for example, at 10 min. These in situ XRD results strongly support the in situ XPS findings, which together reveal a clear and rapid structural transformation of the catalyst under reaction conditions, that is, from CuO to metallic Cu within a short pre‐activation timescale.

STEM analysis was further conducted to investigate the morphology of Cu/Fe_2_O_3_ before and after the reaction. Prior to the reaction, the sample exhibits a morphology as loose nanoparticles without clear boundaries or intact interfaces (Figure S18a). As shown in Figure S18b, the lattice fringe is 0.25 nm, corresponding to the characteristic (002) plane of CuO or the (311) plane of Fe_2_O_3_ [50, 53]. Elemental mapping reveals that Fe, Cu, and O are homogeneously distributed within the framework (Figure S18c–g). No separate phases or aggregation are observed, indicating an atomic mixture of Cu and Fe within the metal oxide framework.

The STEM image of Cu/Fe_2_O_3_ after a 1‐h reaction under optimized conditions was acquired for comparison (Figure 3e–g and Figures S19, S20). There is a significant difference in morphology and element mapping. Many nanoparticles with clear edges can be observed (Figure S19a). Element mapping further reveals that Cu is mainly distributed on these newly formed nanoparticles, while Fe and O are primarily distributed around these nanoparticles (Figure 3e and Figure S19). A distinct interface is observed between Fe and Cu species (Figure 3e). The lattice fringe on the newly formed nanoparticles is 0.21 nm, corresponding to the characteristic (111) planes of metallic Cu (Figure 3f) [54, 55]. The average particle size of these Cu nanoparticles is determined to be 40 nm (Figure 3g). Careful inspection reveals that numerous small nanoparticles are distributed around the larger nanoparticle (Figure S20a). Elemental mapping confirms that the smaller nanoparticles are Fe_2_O_3_, while the larger nanoparticle is identified as metallic Cu (Figure S20b). The lattice fringe of 0.25 nm in the small nanoparticle corresponds to the characteristic (311) planes of Fe_2_O_3_ (Figure S20c) [53]. The average particle size of these Fe_2_O_3_ nanoparticles is determined to be 13.5 nm (Figure S20d), and they aggregate together to support the larger Cu nanoparticles, like a core‐shell structure. These results are consistent with the XRD and XPS findings, indicating that CuO is reduced to metallic Cu while Fe_2_O_3_ remains in an oxidized state during the reaction. The driving force behind the reduction of Cu species was also investigated, as detailed in Figure S21 and Note S7. Furthermore, STEM analysis was performed on Cu/Fe_2_O_3_ after 18 h of reaction (Figure S22). Compared to the sample after 1 h of reaction, a modest increase in particle size from 40 to 49 nm is observed, suggesting that sintering is likely the primary factor contributing to activity deactivation.

### 2.3 Mechanism of IR‐Driven Cu/Fe_2_O_3_ Catalyst

The role of IR photons in this ethanol dehydrogenation reaction was first studied. The light absorption capability of Cu/Fe_2_O_3_ was monitored as shown in Figure S23 and detailed in Note S8, revealing a broad light response extending up to 2500 nm in the IR region.

Transient absorption spectroscopy (TAS) was then conducted to analyze the photon excitation, charge carrier dynamics, and energy transfer processes under IR light illumination (Figure 4a,b and Figure S24). The photon‐induced charge carriers (electrons and holes) in FeO_x_ under IR of 800 nm excitation were initially monitored (Figure S24a). The TAS spectra reveal a broad positive absorption region centered around 650 nm and another positive absorption region below 500 nm extending to 450 nm. To identify the nature of these excited states, methanol vapor as a known photohole scavenger was introduced (Figure S24b) [56]. The presence of methanol enhances the 600–700 nm region intensity while suppressing the 450–500 nm region intensity (Figure 4a and Figure S24b), indicating that the former corresponds to photoelectrons and the latter to photoholes.

**FIGURE 4:**
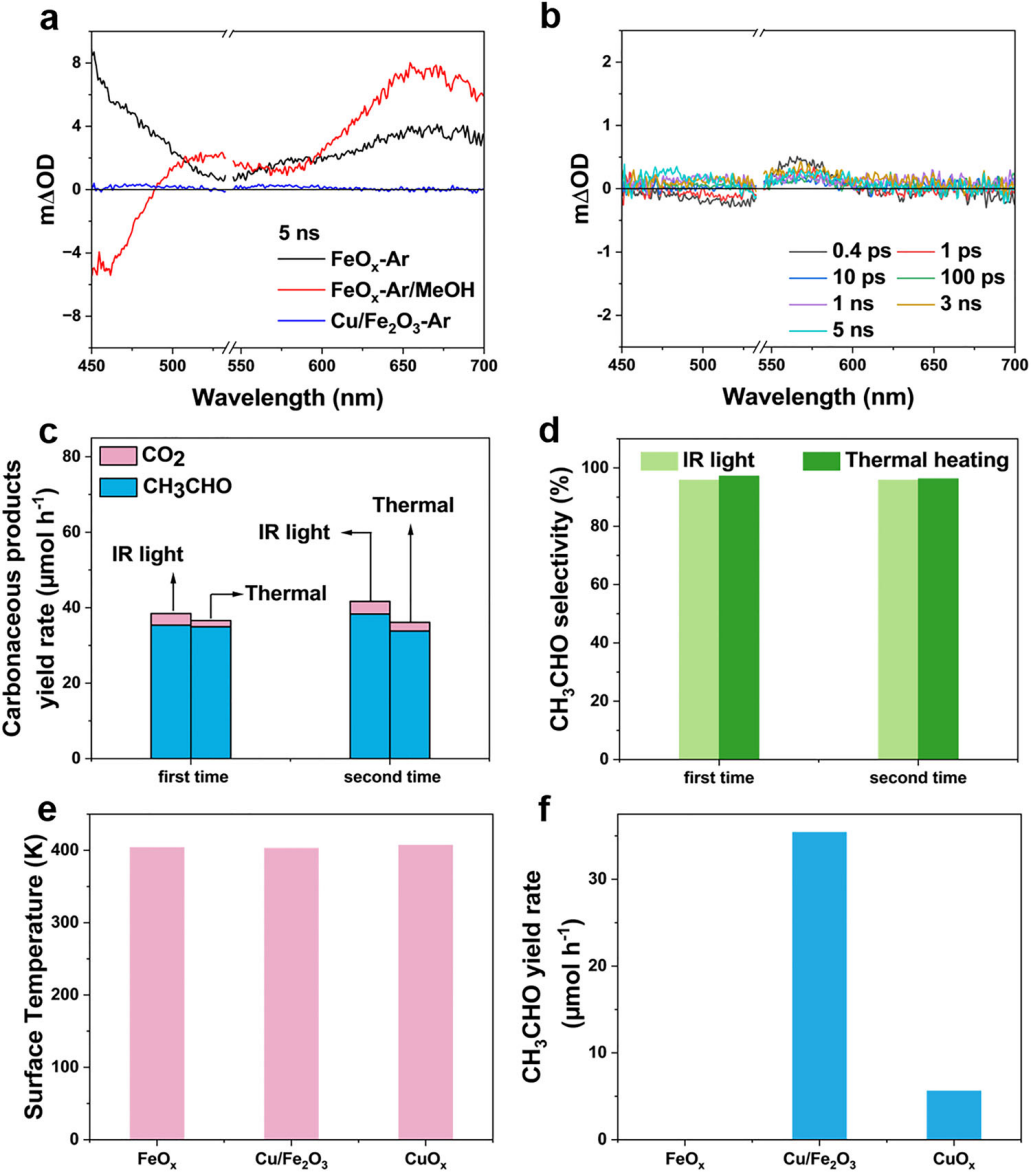
The function of IR light in the acetaldehyde synthesis. (a) TAS spectra at 5 ns after excitation for FeO_x_ under argon, FeO_x_ under argon with methanol vapor, and Cu/Fe_2_O_3_ under argon. (b) fs‐ns TAS spectra of Cu/Fe_2_O_3_ under an argon atmosphere. (Excitation wavelength: 800 nm. Excitation energy: 0.66 mJ cm^−2^ for FeO_x_ and Cu; 1.1 mJ cm^−2^ for Cu/Fe_2_O_3_). (c) The comparison of carbon containing products under IR light irradiation and by thermal heating (403 K) under dark condition. (d) The comparison of CH_3_CHO selectivity under IR light irradiation or thermal heating (403 K) under dark condition. (e) Surface temperature (Ts) over FeO_x_, Cu/Fe_2_O_3_ and CuO_x_ under identical IR light irradiation. (f) The CH_3_CHO yield rates over FeO_x_, Cu/Fe_2_O_3_ and CuO_x_ under identical IR light irradiation (reaction conditions: 30 mL min^−1^ Ar carrier gas, 300 W Xe (700 nm< *λ *<2500 nm), 1037 mW cm^−2^, 60 mL EtOH/H_2_O (1:1) in the gas bubbler).

Upon incorporation of Cu species, the TAS spectra of Cu/Fe_2_O_3_ change dramatically compared to pristine FeO_x_ (Figure 4a). Both photon‐induced absorption regions (450–500 nm and 600–700 nm) disappear at 5 ns, suggesting that photoelectrons and photoholes generated in Fe_2_O_3_ rapidly recombine at the Cu nanoparticle center, thus releasing energy as phonons rather than participating in photon‐driven catalysis. Figure 4b further elucidates that such recombination takes place even at 1 ps.

Given that Cu nanoparticles are known to exhibit localized surface plasmon resonance (SPR) effect [57, 58], we also investigated their charge carrier dynamics using fs TAS (Figure S24c). A strong photon‐induced absorption at ∼565 nm was observed as early as 0.1 ps, indicating hot carrier generation. However, this signal decays rapidly and nearly vanishes by 10 ps, suggesting a fast recombination of charges generated over Cu by the SPR. Careful inspection of the fs‐ns TAS spectra of Cu/Fe_2_O_3_ also reveals a transient photon‐induced absorption feature around 565 nm (0.4 ps after excitation, Figure 4b). Again, this signal diminishes rapidly and has no contribution to the characteristic absorption of photon‐generated carriers in Fe_2_O_3_ (450–500 nm for photoholes and 600–700 nm for photoelectrons, as shown in Figure 4a,b). This observation suggests that the photon‐induced carrier generated on Cu recombine quickly and cannot transfer to Fe_2_O_3_. Overall, this systematic TAS investigation strongly indicates that IR photons contributing thermal effects through phonons released from charge carrier recombination. Other possible pathways for IR‐driven thermal effects are discussed in Note S9, which further supports that phonons in our system arise predominantly from this indirect process. This finding highlights a promising photon‐to‐phonon pathway for initiating efficient photothermal catalysis driven solely by photons, and potentially by solar energy [58, 59].

Then, a modified Harrick Praying Mantis reaction cell was employed to further elucidate the function of IR photons in this reaction (detailed information is provided in the Methods section in Supporting Information, and the reactor's structure is shown in Figure S25). When exposing to IR light irradiation, the surface temperature (Ts) of the Cu/Fe_2_O_3_ in the fundamental study can reach 403 K (lower than the optimized temperature). Under these conditions, a CH_3_CHO yield rate of ca. 35 µmol h^−1^ and a H_2_ yield rate of ca. 33 µmol h^−1^ can be achieved without external heating, with a selectivity to CH_3_CHO of ca. 96% (Figure 4c,d, Figure S26). Subsequently, a reference thermocatalytic experiment was conducted without light irradiation. Interestingly, the reaction activity, in terms of CH_3_CHO yield rate (ca. 35 µmol h^−1^), H_2_ yield rate (ca. 34 µmol h^−1^), and CH_3_CHO selectivity (ca. 97%), driven by thermal energy in the absence of IR light, is similar to that observed under IR light irradiation. This comparison experiment has been repeated again to ensure the reliability of the results. These results suggest that the primary driving force for this reaction is thermal energy. It indicates that the IR photons absorbed by the Cu/Fe_2_O_3_ heat the catalyst, raising the temperature of the localized region on the catalysts for the acetaldehyde synthesis reaction.

Using the same reactor, the Ts of pure FeO_x_ and CuO_x_ are monitored to be 404 and 407 K, respectively, under similar reaction conditions to those used for the Cu/Fe_2_O_3_ catalyst (Figure 4e). These temperatures are very close to that of Cu/Fe_2_O_3_ (403 K), indicating a similar IR photon‐to‐thermal conversion process. While a significant difference in acetaldehyde production is observed (Figure 4f), with no acetaldehyde production over FeO_x_ and only a small amount of production over CuO_x_. The chemical states of Fe and Cu in the FeO_x_ and CuO_x_ post‐reaction were further examined (Figure S27). It is noteworthy that both FeO_x_ and CuO_x_ exhibit similar chemical states to their counterparts in the Cu/ Fe_2_O_3_. The Fe species in FeO_x_ remains in the Fe^3+^ state with no observable changes pre‐ and post‐reactions. In contrast, the Cu in CuO_x_ undergoes a reduction from Cu^2+^ to Cu(0) after the reaction. This result, together with the activity observed among these three samples, suggests the synergistic interactions between Fe and Cu in the Cu/Fe_2_O_3_ catalyst. Furthermore, it is reasonable to guess that Cu serves as the primary catalytic center, while Fe_2_O_3_ likely acts as a promoter. To verify the promoter role of Fe_2_O_3_ in the reaction, an additional control experiment was performed, as presented in Figure S28a and discussed in Note S10.

### 2.4 Interaction Between Catalysts and Reactants

First, the impact of the acidity following the introduction of Fe sites was assessed by NH_3_‐TPD experiments (Figure S29). Notably, a desorption peak spanning 130°C–300°C, with a maximum at approximately 180 °C, is observed for Cu/Fe_2_O_3_, whereas the sample CuO_x_ exhibits an unnoticeable desorption peak. This NH_3_ desorption peak within this temperature range corresponds to Lewis acid sites [60, 61], which can be quantified at 53 µmol g^−1^. Therefore, Fe sites function as Lewis acid sites in the catalyst.

To investigate the effect of these additional Lewis acid sites on ethanol adsorption, a dedicated EtOH/H_2_O‐TPD‐MS experiment was conducted to monitor the in situ adsorption behavior of Cu/Fe_2_O_3_ under the actual reaction environment (Figure S30 and Note S11). As shown in Figure S30a, the characteristic *m/z* = 18 signal for H_2_O is clearly detected, confirming that H_2_O adsorption occurs on the catalyst surface. Importantly, characteristic signals corresponding to EtOH (*m/z* = 45, 31) and CH_3_CHO (*m/z* = 43, 29) are also observed, indicating that ethanol adsorption is not fully suppressed by the presence of water. Enlarged views of these signals are provided in Figure S30b–d for clarity. A careful comparison of the EtOH‐related signals (*m/z* = 45 and 31) reveals significantly higher intensities for Cu/Fe_2_O_3_ than for CuO_x_ (Figure S30b,c). This shows that Fe^3+^ as Lewis acid sites enhances ethanol adsorption even in the presence of H_2_O vapor. A similar trend is observed for the CH_3_CHO‐related signals (*m/z* = 43 and 29), with Cu/Fe_2_O_3_ showing consistently stronger responses than CuO_x_ (Figure S30b,d). To more accurately represent the adsorption strength, the *m/z* = 29 signal (the most intense fragment of CH_3_CHO) was integrated (Figure S30d). Because the baselines of the two samples differ, the integrated peak area provides a more reliable comparison. Cu/Fe_2_O_3_ exhibits a clearly larger peak area than CuO_x_, indicating enhanced adsorption. Moreover, the desorption peak temperature of CH_3_CHO on Cu/Fe_2_O_3_ is approximately 20°C lower than that on CuO_x_, suggesting that the Fe^3+^ Lewis acid sites facilitate ethanol dissociation. As the adsorption process in this EtOH/H_2_O‐TPD‐MS experiment occurs under conditions closely resembling the actual reaction environment, the observation clearly suggests that Fe^3+^ Lewis acid sites promote both ethanol adsorption and dissociation.

To further investigate the role of Fe and Cu during the ethanol dehydrogenation reaction, in situ DRIFTS measurements were carried out (Figure 5a–f). The catalyst was pre‐activated under EtOH/H_2_O vapor flow for 1 h to allow the structural transformation to reach completion before measurements. This approach ensures that the subsequent analysis captures a surface chemistry profile representative of the steady‐state catalytic environment. The adsorption behavior of ethanol was monitored at room temperature over Cu/Fe_2_O_3_, FeO_x_, and CuO_x_. In the spectral region of 3500–2500 cm^−1^ (Figure 5a–c), all three samples exhibit similar absorption features. The bands at 2974 and 2898 cm^−1^ are attributed to the asymmetric and symmetric C–H stretching vibrations of the methyl (CH_3_) group, corresponding to either molecularly adsorbed ethanol (CH_3_CH_2_OH*) or dissociatively adsorbed ethoxy species (CH_3_CH_2_O*) [62, 63]. A progressive increase of characteristic infrared absorption signals is observed over time (0–20 min) for all three samples at room temperature. A more detailed analysis in the 2000–789 cm^−1^ region (Figure 5d–f) reveals distinct spectral features among the three samples. At room temperature on CuO_x_ (Figure 5f), a prominent band at 1051 cm^−1^ is observed, corresponding to the ν(C–O) stretching vibration in ethoxy species, along with minor bands at 1395 cm^−1^ (δ*_s_* (CH_3_)) and 1246 cm^−1^(δ*_s_* (OH)) [63, 64]. In contrast, while FeO_x_ exhibits similar minor bands at 1395 and 1246 cm^−1^, the ν(C–O) stretching shifts slightly to 1064 cm^−1^(Figure 5e), likely due to differences in the interaction strength of ethoxy species with the respective surfaces [64, 65]. Interestingly, Cu/Fe_2_O_3_ (Figure 5d) retains both ν(C–O) stretching bands from CuO_x_ and FeO_x_ at 1051 and 1064 cm^−1^, respectively, indicating the coexistence of adsorption characteristics from both components. These results suggest that ethanol can be preactivated via O─H bond cleavage to form ethoxy species on the catalyst surface at room temperature. Subsequently, the temperature was raised to 513 K to simulate the reaction under the optimized reaction conditions, and the corresponding DRIFTS were recorded.

**FIGURE 5:**
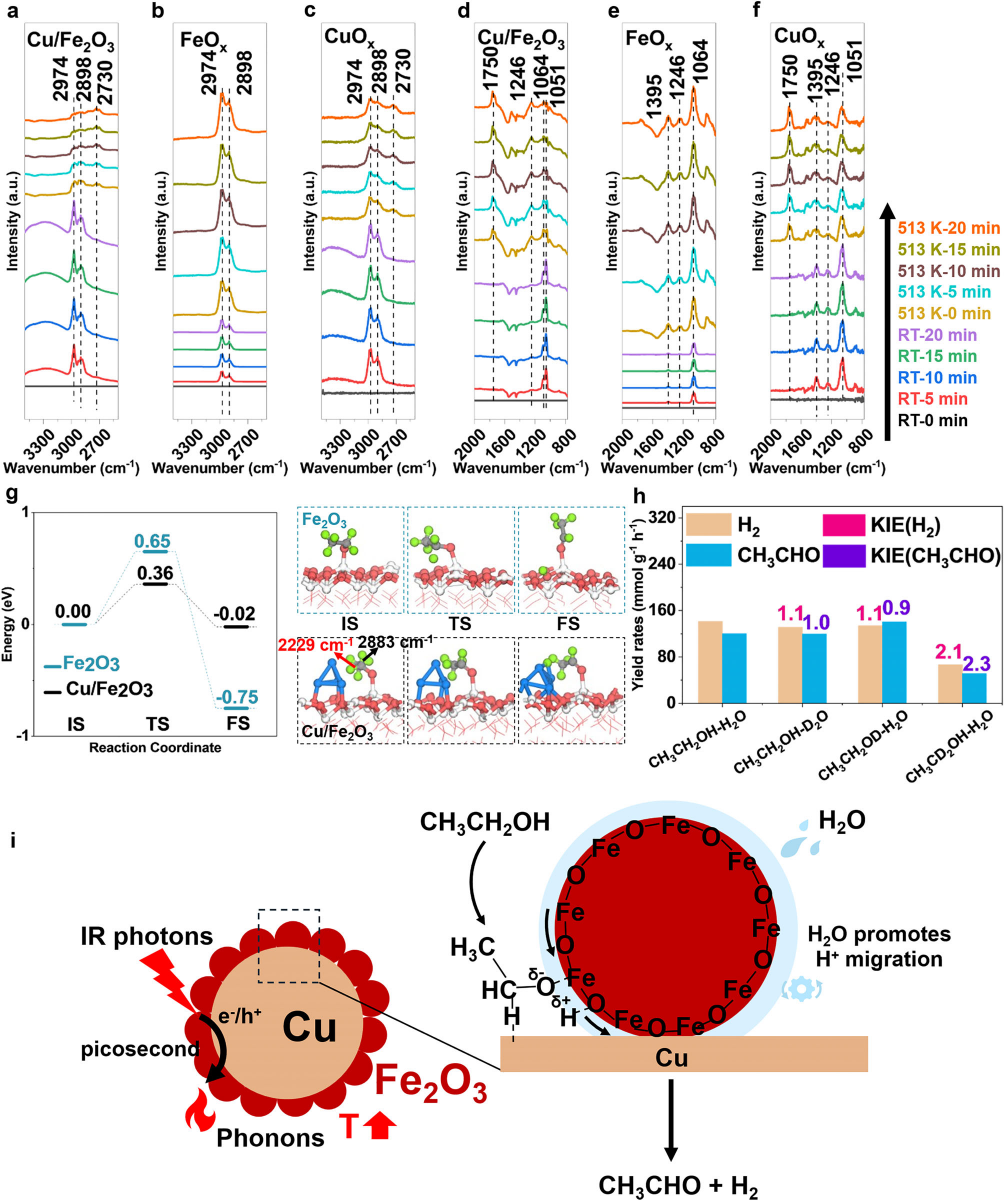
The surface reaction mechanism of the catalyst. (a–c) The in situ DRFITS spectra of Cu/Fe_2_O_3_, FeO_x_ and CuO_x_ in the region of 3500–2500 cm^−1^ under Ar flow with EtOH/H_2_O vapor from room temperature (RT) to 513 K; (d–f) the in situ DRFITS spectra of Cu/Fe_2_O_3_, FeO_x_ and CuO_x_ at the region of 2000–770 cm^−1^ under Ar flow with EtOH/H_2_O vapor from room temperature to 513 K (*Note*: The intensity scales vary across the figures to allow zoomed‐in views). (g) Calculated energy profiles for ethoxy group dehydrogenation to acetaldehyde on the Cu/Fe_2_O_3_ and Fe_2_O_3_ surfaces, and the corresponding structures of the key states are also shown (red: O atoms; green: H atoms; grey: C atoms; white: Fe atoms; blue: Cu atoms). (h) The KIE effect investigation using CH_3_CH_2_OH/H_2_O, CH_3_CH_2_OH/D_2_O, CH_3_CH_2_OD/H_2_O, and CH_3_CD_2_OH/H_2_O. (Reaction conditions: 20 mg Cu/Fe_2_O_3_ catalyst, 60 mL min^−1^ Ar carrier gas, 300 W Xe lamp (700 nm< *λ *<2500 nm, 1226 mW cm^−2^), ethanol/water (molar ratio: 2:1), 0.01 mL min^−1^ liquid injection rate, no external heating. KIE_H2_ = k(H_2_)__nonisotope_/k(H_2_)__isotope_, KIE_CH3CHO_ = k(CH_3_CHO)__nonisotope_/k(CH_3_CHO)__isotope_, k is the production rate). (i) The proposed reaction scheme over Cu/Fe_2_O_3_ under IR irradiation.

During the heating period at 513 K, the intensity of the IR bands corresponding to the CH_3_CH_2_OH*/CH_3_CH_2_O* gradually decreases on both Cu/Fe_2_O_3_ and CuO_x_ (Figure 5a,c,d,f). Furthermore, a new band emerges at approximately 1750 cm^−1^ on both Cu/Fe_2_O_3_ and CuO_x_ (Figure 5d,f), which is characteristic of ν(C═O) stretching vibration in acetaldehyde [63, 66], indicating the conversion of ethanol to acetaldehyde. This is further supported by the new appearance of a band around 2730 cm^−1^ (Figure 5a,c), attributed to the ν(C–H) stretching vibration in acetaldehyde [66]. In contrast, these features are barely observed on FeO_x_ (Figure 5b,e). These results suggest that Cu serve as the active site instead of Fe species for ethanol dehydrogenation to acetaldehyde and hydrogen.

Then the intensity ratio of the 2974 cm^−1^ band monitored at 513 K (20 min) and room temperature (20 min) was used to half‐quantify the ethanol conversion. It is 0.20 for Cu/Fe_2_O_3_ and 0.57 for CuO_x_ (Figure 5a,c), indicating a more efficient ethanol conversion on Cu/Fe_2_O_3_. Additionally, the ratio of the 1750 cm^−1^ band (acetaldehyde) to the 1051 cm^−1^ (ethoxy) after 20 min at 513 K is 1.8 for Cu/Fe_2_O_3_ and 1.4 for CuO_x_, suggesting a higher acetaldehyde yield on Cu/Fe_2_O_3_. This rough estimation is further supported by ethanol‐TPD measurements above, which confirm a higher ethanol adsorption capacity on Cu/Fe_2_O_3_ compared to CuO_x_. The absence of Fe in CuO_x_ results in a lower conversion rate, highlighting the importance of both Fe and Cu species in Cu/Fe_2_O_3_. So herein, Cu serves as the active center, and Fe acts as a promoter, enabling a synergistic effect that enhances the catalytic performance, consistent with the results from ethanol dehydrogenation tests.

To gain deeper insights into the synergistic effect at the confined interface, density functional theory (DFT) calculations were also performed to investigate the dehydrogenation of ethoxy groups on the Cu/Fe_2_O_3_ and Fe_2_O_3_ surfaces (Figure 5g). The model information is shown in Figure S31 with a detailed description in the Methods section. The calculated results show that the stretching frequency of the *α*‐C─H bond in the ethoxy group adjacent to interfacial Cu (2229 cm^−1^) is significantly lower than that of the *α*‐C─H bond farther away from Cu (2883 cm^−1^), indicating that the presence of Cu at the interface can effectively weaken the *α*‐C─H bond. Consistently, the activation energy for ethoxy dehydrogenation at the Cu/Fe_2_O_3_ interface is 0.36 eV, much lower than that on pure Fe_2_O_3_ (0.65 eV). The electronic interaction has also been investigated (Note S12). Supported by the in situ DRIFTS results and DFT calculations, the synergistic activation mechanism on the Cu/Fe_2_O_3_ interface has been further solidified.

Next, systematic KIE investigations were performed to identify the rate‐determining step (Figure 5h). Four runs were undertaken to examine KIE effects, such as CH_3_CH_2_OH/H_2_O, CH_3_CH_2_OH/D_2_O, CH_3_CH_2_OD/H_2_O and CH_3_CD_2_OH/H_2_O. When using CH_3_CH_2_OH/D_2_O and CH_3_CH_2_OD/H_2_O, the KIE_H2_ (1.1 and 1.1) and KIE_CH3CHO_ (1.0 and 0.9) are close to 1, indicating that O─H bond activation in water or ethanol is not the rate‐determining step. It should be noted that when CH_3_CD_2_OH/H_2_O was used as the feedstock, the KIE_H2_ and KIE_CH3CHO_ values are 2.1 and 2.3, respectively. These significant isotope effects suggest that C─H bond cleavage in the CH_2_ group of ethanol is the rate‐determining step. Furthermore, the corresponding isotope distribution in the product, acetaldehyde, was analyzed, as shown in Figure S32. The molecular weight of CH_3_CHO is 44. When using CH_3_CH_2_OH/H_2_O as the feedstock, the dominant m/z peaks are 44, 43, 42, 41, and 40, which are CH_3_CHO and its fragments. While using CH_3_CH_2_OH/D_2_O and CH_3_CH_2_OD/H_2_O, there is a minor peak at 45 (likely due to slight D‐H exchange), and the most intense peak is still at 44, indicating that the major product is CH_3_CHO. When CH_3_CD_2_OH/H_2_O was used as the feedstock, the dominant m/z peaks shift by 1, with the most intense peak at 45, corresponding to CH_3_CDO. This observation further confirms the reaction pathway via ethanol dehydrogenation (CH_3_CH_2_OH→CH_3_CHO+H_2_).

Given that the O─H bond in H_2_O has a higher bond energy (∼492 kJ/mol) than the C─H bond energy (∼312 kJ/mol) in the CH_2_ group of ethanol [67, 68], if the reaction process involves the activation of the O─H bond in H_2_O, it should affect the reaction rate. Based on the isotope distribution results mentioned above, H_2_O acts as a promoter rather than a reactant in this ethanol dehydrogenation reaction. Two additional facts further support this claim: i). A much higher temperature (573 to 1073 K) is required in the ethanol/water reforming reaction to activate H_2_O and to generate hydroxyl radicals to participate in the reaction [28]. In contrast, water was not involved in the reaction of formaldehyde synthesis using methanol as feedstock at relatively low temperatures, for example, 383 K [69]. ii). The addition of water has been observed with a promoting effect in similar processes [69, 70]. The presence of water can facilitate methanol deprotonation by stabilizing the H atom from the O─H bond to form a methoxy group. Furthermore, water‐assisted migration of protons, or “proton hopping”, has been observed on FeO films [71], which is believed to facilitate the mass transfer of protons, allowing them to recombine and generate H_2_. Therefore, water acts as a promoter in our system reaction, facilitating the conversion of ethanol to form acetaldehyde and migration of protons [69, 70].

Based on the above findings, a reaction mechanism is proposed to illustrate the synergistic effect between Cu and Fe_2_O_3_ in ethanol activation (Figure 5i). When excited by IR photons, the charge carriers initially generated rapidly decay at picosecond timescale into phonons, leading to a localized temperature increase. After a short induction period, the copper component of the catalyst undergoes a rapid in situ transformation into metallic Cu nanoparticles surrounded by small Fe_2_O_3_ particles, creating a highly active Cu/Fe_2_O_3_ interface. In parallel, ethanol molecules absorb onto the interface, forming ethoxy species. Then the interface selectively facilitates the decomposition of ethoxy species into acetaldehyde. Herein, the Fe^3+^ Lewis acid sites enhance ethanol adsorption and promote O─H bond cleavage. Importantly, the metallic Cu nanoparticles activate the C─H bond in the CH_2_ group of the adsorbed CH_3_CH_2_O*. Following C─H bond scission, the resulting CH_3_CHO desorbs from the surface, and the remaining protons transport to recombine together for H_2_ generation.

## 3 Conclusion

In summary, a highly efficient sunlight IR‐driven catalyst, Cu/Fe_2_O_3_, has been developed for acetaldehyde synthesis from bioethanol. Under indoor IR irradiation, the catalyst achieves a notable initial CH_3_CHO yield of 237 mmol g^−1^ h^−1^ with ∼97.7% selectivity, accompanied by nearly stoichiometric green H_2_ production as the sole byproduct. Notably, this is the first IR photon‐driven catalyst capable of simultaneously producing both a valuable carbon‐containing product and hydrogen from bioethanol. Using H_2_ production as a reference, the TON and initial TOF (based on the weight of active component) exceed all reported IR‐driven systems by at least one order of magnitude and perform competitively with energy‐intensive UV–vis photocatalytic ethanol conversion systems. When using CH_3_CHO production as the index, its productivity and selectivity, together with the TON and initial TOF (based on the number of surface active sties), again place it among the leading catalysts when compared with conventional thermocatalytic ethanol dehydrogenation processes operated at temperatures up to 573 K. Fundamentally, this work establishes a novel IR photon‐to‐phonon conversion pathway, in which excited photoelectrons and photoholes in Fe_2_O_3_ recombine at metallic Cu centers and hot carriers generated via the Cu SPR effect decay rapidly, collectively releasing phonons at ps timescale to drive localized thermocatalysis. Additionally, a synergistic effect is observed at the in situ formed Cu/Fe_2_O_3_ interface: Fe^3+^ as Lewis acid sites promote dissociative ethanol adsorption, while metallic Cu facilitates hydrogen abstraction from the CH_2_ group of the ethoxy intermediates, leading to acetaldehyde formation. This work represents a significant advancement in natural sunlight‐driven synthesis of valuable chemicals from biomass derivatives.

## Author Contributions

**Xiyi Li**: investigation, writing – original draft, writing – review and editing, formal analysis, validation. **Jiangting Zhao**: investigation, writing – review and editing, formal analysis, validation. **Junjun Guo**: investigation, formal analysis, funding acquisition, writing – review and editing. **Ze‐Kai Yu**: software, investigation, formal analysis, writing – review and editing. **Enqi Chen**: investigation, formal analysis. **Qiong Liu**: investigation, formal analysis. **Tieou Wang**: investigation, formal analysis. **Asterios Gavriilidis**: investigation, validation, formal analysis, writing – review and editing. **Xue‐Qing Gong**: investigation, software, formal analysis, writing – review and editing. **Yang Lan**: writing – review and editing, supervision, resources, funding acquisition, formal analysis, project administration, conceptualization. **Junwang Tang**: conceptualization, formal analysis, supervision, project administration, resources, writing – review editing, writing – original draft, funding acquisition.

## Conflicts of Interest

The authors declare no conflicts of interest.

## Supporting information




**Supporting File 1**: anie74037‐sup‐0001‐SuppMat.docx.

## Data Availability

The data that support the findings of this study are available from the corresponding author upon reasonable request.
