# Supplementary material for: Natural Sunlight IR‐Driven Highly Efficient Synthesis of Acetaldehyde From Bioethanol Over Cu/Fe2O3
Source: Angew Chem Int Ed Engl. 2026 Aug 5;65(40):e9513626. doi: 10.1002/anie.9513626 (PMC13618288; doi:10.1002/anie.9513626)
Supplement: Supplementary file 1 [file ANIE-65-e9513626-s001.docx]

**Supporting Information**

**Natural sunlight IR-driven highly efficient synthesis of acetaldehyde from bioethanol over Cu/Fe_2_O_3_**

**Authors:** Xiyi Li^1 +^, Jiangting Zhao^2 +^, Junjun Guo^2^, Ze-Kai Yu^3^, Enqi Chen^1,2^, Qiong Liu^4^, Tieou Wang^2^, Asterios Gavriilidis^1^, Xue-Qing Gong^5^, Yang Lan^1^*, Junwang Tang^1,2^*

**Methods**

**Materials**

Chemicals for materials synthesis: Ce(NO_3_)·6H_2_O (ALDRICH, 99%), Mn(NO_3_)_2_·4H_2_O (SIGMA-ALDRICH, ≥97.0%), Co(NO_3_)_2_·6H_2_O (ACROS, 99+%), Cu(NO_3_)_2_·2.5H_2_O (ALDRICH, ≥99.99%), Ni(NO_3_)_2_·6H_2_O (SIGMA-ALDRICH, ≥97.0%), Zn(NO_3_)_2_·6H_2_O (Thermos scientific, 98%), Fe(NO_3_)_3_·9H_2_O (SIGMA-ALDRICH, ≥98%), Citric Acid Anhydrous (Fisher Scientific, ≥99.5%).

Reactants: Ethanol (Fisher Chemical, ≥99.8%), Deionised water (18.2 MΩ·cm).

**Catalyst synthesis**

The synthesis of dual metal oxides was conducted using the citric acid combustion method reported previously.^[1]^ The process began with the combination of 2 g first metal precursor, 2 g second metal precursor, and 0.8 g citric acid. A small volume of deionized water was then added to this mixture. The mixture was stirred on a hot plate until the formation of a gel. This gel was subsequently calcined in air at a temperature of 350°C through a ramp of 10 °C /min for a duration of 3 hours. The resulting products were then grounded to complete the process. A series of catalysts such as Cu/CoO_x_, Cu/MnO_x_, Cu/NiO_x_, Co/FeO_x_, Cu/Fe_2_O_3_, Mn/FeO_x_, Ni/FeO_x_, Ce/MnO_x_, Ni/MnO_x_, and Ni/ZnO_x_ were prepared.

The synthesis of Cu/Fe_2_O_3_ with different Fe and Cu molar ratios followed the similar procedure. Typically, it started with the combination of Fe(NO_3_)_3_·9H_2_O and Cu(NO_3_)_2_·2.5H_2_O in varying molar proportions, such as 1:0, 3:1, 2:1, 1:1, 1:2, 1:3, and 0:1. In addition, an appropriate amount of citric acid was added to this mixture to achieve a 1:1 molar ratio with the mixture. A small volume of deionized water was then added to this mixture. The concoction was then agitated on a hot plate at around 95°C until it transformed into a gel. The gel was subsequently subjected to a calcination process in an air atmosphere at a temperature of 350°C, with a ramp rate of 10 °C /min for a duration of 3 hours. The end product was then grinded.

**IR-driven acetaldehyde synthesis**

The preliminary catalysts evaluation for the IR-driven acetaldehyde synthesis reaction was carried out using a flow reactor (Figure S1 and Note S1). 20 mg of catalyst powder was dispersed in 200 mL of water via sonication and filtered on a glass fibre membrane (diameter 37 mm, pore size 0.22 µm) to obtain a uniform catalyst layer. The membrane was dried and fixed in the centre of the reactor with a stainless steel ring. Then, the reactor was sealed with a rubber O-ring and a stainless steel cover. The top of the cover was a quartz window with a diameter of 3 cm, which allowed the IR light irradiation. An argon (BOC, 99.999%) carrier gas, at a rate of 30 mL min^-1^ controlled by mass flow controller (Bronkhorst), was fed through a 60 mL mixture of EtOH/H_2_O (molar ratio, 1:1) at room temperature, transporting ethanol and water to the reactor. A 300 W Xe lamp (Perfectlight. Co., microsolar 300), equipped with a 700 nm long-pass filter (700 nm < λ < 2500 nm), served as the IR light source. During the reaction, the outlet of the reactor was first connected to a collection bottle containing 100 mL of H_2_O immersed in an ice bath to condense and absorb liquid products. The outlet tubing of the collection bottle was then connected to the inlet of a gas chromatograph (Shimadzu GC-2010 Pro) equipped with a barrier discharge ionization detector (BID-2010 Plus). The Shimadzu GC system included two 500 μL sampling loops, allowing injection of gas samples into separate analytical channels. The gas analysis setup employed two 10-port valves operating in a backflush configuration:

Valve 1 directed the sample to a pre-column where only light gases (Ar, H_2_, O_2_, N_2_, CH_4_, CO) passed through to a Molesieve 5A column for separation and detection. All other components were backflushed to the vent. This channel enabled quantification of gaseous products (H_2_, CH_4_, CO) from the reaction.

Valve 2 injected the same sample to a second pre-column, allowing gases (Ar, H_2_, O_2_, N_2_, CH_4_, CO, CO_2_, C_2_H_4_, C_2_H_6_) to enter an Rt-Q-Bond column for separation of CO_2_, C_2_H_4_, and C_2_H_6_ from light gases, while other components were backflushed to the vent. This channel enabled quantification gaseous products (CO_2_, C_2_H_4_, and C_2_H_6_) from the reaction.

After one hour of reaction, the liquid products accumulated in the ice bath were collected for analysis. A PoraPlot Q column connected to the flame ionisation detector (FID) in the Agilent 7820 gas chromatograph was used to separate and analyse the liquid-phase products, including acetaldehyde, ethanol, acetone, ethyl acetate. An autosampler ensured consistent injection volumes into the column. After sampling, the condensation collection bottle was cleaned and refilled with fresh water to prepare for the next one-hour reaction run.

For the investigation of the influence of irradiance, GHSV and EtOH/H_2_O molar ratios on the performance of Cu/Fe_2_O_3_, the reaction was carried out in the same reactor with modification of the liquid feed. As shown in Figure S7, argon was introduced into the reactor at a setting flow rate, while an ethanol-water mixture (EtOH: H_2_O) was supplied at a controlled flow rate using a syringe pump (10 mL). An evaporator operating at approximately 200°C, served to vaporize the liquid mixture, allowing it to mix with the carrier gas before reaching the reactor. The rest part of the reaction system was the same as mentioned above. Mass spectra in KIE experiments were measured using an Agilent 8890-5977 GC-MS equipped with a PoraPlot Q-HT column, which connected the autosampler injection port to the MS detector. For the investigation of stability under optimised reaction conditions, the syringe pump was switched to a larger (50 mL) to reduce refill frequency, which introduced fluctuations in feed rates.

For the investigation of photon-to-thermal effect shown in Figure 4c-f, a modified Harrick Praying Mantis reaction cell was used. The schematic of the cell was shown in Figure S25. An argon carrier gas, at a rate of 30 mL min^-1^, was fed through a 60 mL mixture of EtOH/H_2_O (molar ratio, 1:1) in a gas bubbler, transporting ethanol and water to the reactor. A glass fibre membrane (diameter 6 mm) with a thin catalyst layer (ca. 0.63 mg) was cut from the bigger membrane prepared using the method mentioned above, and positioned at the centre of the sample cup. Below the catalyst membrane, the cup was filled with sand and quartz wool. A thermocouple and a heating rod were inserted at the bottom of the sample cup to regulate the heat input and monitor the base temperature (T_b_). Another K-type thermocouple (0.5 mm) contacted perpendicularly the catalyst surface to monitor the surface temperature (T_s_). A 300 W Xe lamp, equipped with a 700 nm long-pass filter (700 nm < λ < 2500 nm), served as the IR light source (1037 mW cm^-2^). Cool water, set at 15°C, was used to maintain a low temperature on the other reactor’s wall, preventing the rubber O-ring from overheating. Under pure heating and dark conditions, a heating element was used to heat the catalyst in a dark environment until the T_s_ match that under light irradiation to ensure a fair comparison. The analysis of products used the set-up as mentioned above.

Light irradiance was calibrated using a thermopile detector (919P-003-10), known for its relatively flat spectral response, which was connected to a power meter (Model 1918-R, Newport). Before measurement, the Xe lamp was operated for over 10 minutes to stabilise its output. The detector was positioned at the similar distance from the light source as the catalyst to ensure accurate intensity readings.

**Natural solar-driven acetaldehyde synthesis**

The outdoor solar-driven acetaldehyde synthesis experiment was conducted in a quartz flow reactor. The reaction system was built as shown in Figure 2a. The catalyst membrane (ca. 40 mm diameter) was prepared using the similar method as mentioned above. The optimised reaction conditions (120 mL min^-1^ Ar carrier gas, 20 mg catalyst, EtOH/H_2_O molar ratio of 2:1, 0.02 ml min^-1^ liquid injection rate) were used for the activity evaluation. An evaporator operating at approximately 200 °C vaporised the liquid mixture, allowing it to mix with the carrier gas before entering the quartz reactor. Aluminon foil was used to cover the edge surface of the quartz reactor to protect the rubber O-ring from direct light irradiation, leaving an area of ca. 7 cm diameter at the centre for light exposure. Due to the low intensity of the sunlight in Autumn, a Fresnel lens was used to concentrate the light, providing appropriate IR photons to reach the catalyst surface. During the reaction, the sunlight’s irradiation angle constantly changed, requiring adjustments to the Fresnel lens position to maintain proper alignment. The irradiance at the beginning of each experimental cycle was recorded for reference, as shown in Figure 2b. It should be noted that the solar irradiance varied during the test period, with occasional clouds blocking the light. Therefore, the total yield of products in each hour, rather than the average yield rate, was used for the activity evaluation. The outlet gas mixture was condensed by passing through 50 mL of water in an ice bath. The gas products were fully collected in a 10 L gas bag, while the liquid products were obtained in the liquid bottle. The gas products were subsequently analysed using a Panna Gas Chromatograph (GC) equipped with a Barrier Ionization Discharge (BID) detector. The liquid products were analysed using a Shimadzu GC with a Flame Ionization Detector (FID). The irradiance was measured using the PL-MW2000 high-power light meter from PerfectLight. At the end of each experimental cycle, the catalyst was stored in the reactor with continuous argon flow. The experiment was conducted during seven sunny periods on Tsinghua University campus, Beijing, China.

**Calculation of EtOH conversion, CH_3_CHO selectivity, H_2_/CH_3_CHO ratio and carbon balance**

$${Con}_{EtOH}=\frac{n_{CH4}+n_{CO}+n_{CO2}+2\times(n_{C2H6}+n_{C2H4}+n_{CH3CHO}{+n}_{CH3COOH}{)+3\times n_{CH3COCH3}+4\times n_{CH3COOCH2CH3}}}{n_{CH4}+n_{CO}+n_{CO2}+2\times(n_{C2H6}+n_{C2H4}+n_{CH3CHO}{+n}_{CH3COOH}{+n}_{C2H5OH(out)}{)+3\times n_{CH3COCH3}+4\times n_{CH3COOCH2CH3}}}\times100\%$$

$${Sel}_{CH3CHO}=\frac{2\times n_{CH3CHO}}{n_{CH4}+n_{CO}+n_{CO2}+2\times(n_{C2H6}+n_{C2H4}+n_{CH3CHO}{+n}_{CH3COOH}{)+3\times n_{CH3COCH3}+4\times n_{CH3COOCH2CH3}}}\times100\%$$

$${Ratio}_{H2/CH3CHO}=\frac{n_{H2}}{n_{CH3CHO}}$$

$$Carbon balance=\frac{n_{CH4}+n_{CO}+n_{CO2}+2\times(n_{C2H6}+n_{C2H4}+n_{CH3CHO}{+n}_{CH3COOH}{+n}_{C2H5OH(out)}{)+3\times n_{CH3COCH3}+4\times n_{CH3COOCH2CH3}}}{2\times n_{C2H5OH(input)}}\times100\%$$

where n is the molar amount of different products obtained for one hour reaction; n_C2H5OH(out)_ is the unreacted ethanol collected in the outlet of one hour reaction; n_C2H5OH(input)_ is the theoretical molar amount of ethanol delivered by the syringe pump for one hour.

**Calculation of turnover frequency (TOF/TON)**

To quantify the number of surface Cu species as they are active sites, the combination of temperature-programmed reduction (H_2_-TPR) measurement and N_2_O oxidation was employed.^[2,3]^ First, the fresh catalyst (30 mg) was subjected to oxidative pretreatment by passing 5% O_2_/He at a flow rate of 20 mL/min through the TPR reactor. The temperature was ramped from 40 °C to 300 °C at 10 °C/min and held at 300 °C for 10 min. Subsequently, the catalyst was reduced using 5% H_2_/Ar under the same ramping conditions (10 °C/min from 40 °C to 300 °C) and held at 300 °C for 1 h. The hydrogen consumption during this step was denoted as Y_1_. After the reduction, the catalyst was cooled to 40 °C under 5% H_2_/Ar and then purged with Ar for 10 min. The sample was then exposed to 1% N_2_O/Ar at a flow rate of 20 mL/min for 60 min to selectively reoxidize surface metallic Cu to Cu_2_O. Residual adsorbed N_2_O was removed by purging with Ar at 40 °C (20 mL/min) for 30 min. A second TPR was subsequently carried out using 5% H_2_/Ar at 20 mL/min with a temperature ramp of 10 °C/min from 40 °C to 350 °C. The hydrogen consumption during this step was recorded as Y_2_.

The H_2_ consumption of Y_2_ reflected to the amount of surface Cu_2_O formed via N_2_O oxidation based upon the equation of Cu_2_O + H_2_ → 2Cu + H_2_O^[3]^. The Cu dispersion D (%) and the number of surface active Cu sites N_Cu_ (mol/g_cat_) were calculated on the basis of the following relationship:

D=2Y_2_/Y_1_

N_Cu_=D×C/M_Cu_

where C was Cu content in the catalyst (%) determined by the ICP-OES (Supplementary Table 1), M_Cu_ was the atomic weight of copper.

Thus, the Cu dispersion (or the ratio of the surface active Cu amount to the total Cu amount) and the number of surface active Cu sties were determined to be 8.3% and 0.64 mmol g_cat_^-1^, respectively. These values enable the calculation of initial turnover frequency normalised to the number of surface Cu atoms (TOF_A_) under the optimised reaction condition. The initial turnover frequency normalised to the Cu weight (TOF_w_) under the same reaction conditions was also calculated. Because the number of surface active sites is generally not reported for photo-driven catalytic systems, the TOF values for these systems were calculated based on the weight of the active component (denoted as TOF_w_, unit: mmol g_M_^-1^ h^-1^). In contrast, the number of surface active sites is widely available for thermocatalysts; therefore, TOF values for thermocatalytic systems were calculated based on the number of surface active sites (denoted as TOF_a_, unit: h^-1^).

$${TOF}_{a}(CH_{3}CHO)=\frac{intial CH_{3}CHO producion rate}{N_{Cu}}=\frac{237 mmol g^{-1} h^{-1}}{0.64 mmol g^{-1}}={370 h}^{-1}$$

$${TOF}_{a}(H_{2})=\frac{intial H_{2} producion rate}{N_{Cu}}=\frac{232 mmol g^{-1} h^{-1}}{0.64 mmol g^{-1}}={363 h}^{-1}$$

$${TOF}_{w}(CH_{3}CHO)=\frac{intial CH_{3}CHO producion rate}{Cu content}=\frac{237 mmol g^{-1} h^{-1}}{49.18 wt\%}={482 mmol g_{Cu}^{-1} h}^{-1}$$

$${TOF}_{w}(H_{2})=\frac{intial H_{2} producion rate}{Cu content}=\frac{232 mmol g^{-1} h^{-1}}{49.18 wt\%}={472 mmol g_{Cu}^{-1} h}^{-1}$$

The turnover number (TON) for this work was calculated based on the 200-hour stability test. The measured H_2_/CH_3_CHO production rates were used to estimate the total amount of H_2_/CH_3_CHO generated during each sampling interval. Note: this represents a conservative estimate, because the production rate decreases during the initial stages; thus, using the measured value at each interval likely underestimated the true amount produced. Because most UV-Vis or IR photocatalysts reported only the weight of active component, and H_2_ was more frequently reported than carbon-containing products (which also vary in type across systems), TON normalised to Cu weight (TON_w_), calculated from H_2_ production, was used for comparison. In contrast, since most thermocatalysts reported the number of surface active sites and ethanol dehydrogenation to CH_3_CHO was widely documented, TON normalised to the number of surface Cu atoms (TON_a_), calculated from CH_3_CHO production, was used for comparison. For systems lacking stability evaluation, TON could only be estimated from the reported reaction period.

$${TON}_{a}(CH_{3}CHO)=\frac{Accumulated amount of CH_{3}CHO}{N_{Cu}*Catalyst mass}=23005$$

$${TON}_{a}\left( H_{2} \right)=\frac{Accumulated amount of H_{2}}{N_{Cu}*Catalyst mass}=20787$$

$${TON}_{w}(CH_{3}CHO)=\frac{Accumulated amount of CH_{3}CHO}{Cu content}={29937 mmol g_{Cu}^{-1}}$$

$${TON}_{w}(H_{2})=\frac{Accumulated amount of H_{2}}{Cu content}={27051 mmol g_{Cu}^{-1}}$$

**Calculation of Solar-to-chemical energy conversion efficiency**

The calculation of energy conversion efficiency was based on the chemical reaction equation (C_2_H_5_OH (g) → CH_3_CHO (g) + H_2_ (g), ∆G° = 39.6 kJ mol^-1^).

$$Energy conversion efficiency =\frac{\Delta G^{\circ}*r}{I*A*t}\times100\%$$

where r was the generation amount of CH_3_CHO in one hour reaction, I was the irradiance of outdoor sunlight, A was the irradiation area, t was the reaction time. For outdoor experiment (the best performance is used for calculation), r was ca. 4.1 mmol, I was 2570 mW cm^-2^, A was ca. 12.56 cm^2^, t was 1 h. The energy conversion efficiency was thus calculated to be 0.14%.

**Catalysts characterisations**

The powder X-ray (XRD) spectra were collected using a Stoe STADI-P instrument with Mo Kα1 (wavelength 0.70930 Å, 50 kV and 30 mA). Ultraviolet-visible diffuse reflectance spectroscopy (UV-Vis DRS) measurements for spectrum were performed by an Agilent Cary UV-Vis-NIR spectrophotometer. XPS Analysis was carried out using an Axis Supra (Kratos Analytical Ltd., UK) with an Al Kα X-ray source and the data was analysed with CasaXPS software calibrated by C 1s peak at 284.8 eV. The element content was measured by an Agilent 5110 Inductively Coupled Plasma Optical Emission Spectroscopy (ICP-OES). STEM imaging was conducted using a FEI Titan Cubed Themis G2 300 with Energy Dispersive-X-Ray (EDX) module.

**In-situ XPS measurement**

In-situ X-ray photoelectron spectroscopy (XPS) measurements were conducted using an AXIS SUPRA spectrometer operating at a base pressure of 2 × 10^-9^ Torr, equipped with a monochromatized Al Kα X-ray source (1486.6 eV) and a 100 μm beam diameter. The system includes a reaction chamber connected to the analysis chamber via an ultra-high vacuum (UHV) transfer arm, allowing sample transfer without air exposure. Catalyst samples were pressed into tablets and placed in a micro quartz holder, which was then introduced into the reaction chamber. To closely replicate the actual reaction environment, the temperature of the heating element was set to 533 K, ensuring that the catalyst surface reached approximately 513 K—matching the optimised reaction conditions. Additionally, the reactants were aligned with those used in actual catalytic activity tests: 120 mL min^-1^ Ar as the carrier gas, an EtOH/H_2_O molar ratio of 2:1, and a liquid injection rate of 0.02 mL min^-1^. The liquid mixture was vaporized using an evaporator maintained at ~473 K and mixed with the carrier gas before entering the reaction chamber. After reaction times of 3, 5, and 10 minutes, the samples were transferred under vacuum to the analysis chamber for XPS measurements. Spectra were collected for Cu 2*p*, Cu LMM, and Fe 2*p* regions to monitor the evolution of surface chemical states. The data was analysed with CasaXPS software calibrated by C 1s peak at 284.8 eV.

**In-situ XRD measurement**

In-situ X-ray diffraction (XRD) measurements were performed using a Rigaku Smartlab diffractometer equipped with Cu Kα radiation and an XRK 900 reactor chamber (Anton Paar). Data were collected over a 2θ range of 10-80° at a scan rate of 50° min^-1^. The catalyst was heated to 513 K at a ramp rate of 25 °C min^-1^ under a flow of Ar (120mL min^-1^). After acquiring the initial diffraction pattern, the gas flow was switched to match the conditions used in catalytic activity tests: 120 mL min^-1^ Ar as the carrier gas, an EtOH/H_2_O molar ratio of 2:1, and a liquid injection rate of 0.02 mL min^-1^. The liquid mixture was vaporised using an evaporator maintained at ~473 K and mixed with the carrier gas before entering the reaction chamber. XRD patterns were recorded every 5 minutes throughout the reaction process to monitor structural evolution in real time.

**TAS measurements**

fs-ns transient absorption spectroscopy (TAS) measurements were performed using a regeneratively amplified Ti: Sapphire laser system (Astrella-Tunable-USP-1K), which generates 800 nm laser pulses with a pulse width 87 fs and a repetition rate 1 kHz. The transient absorption setup was based on a commercially system (Helios, Ultrafast Systems). To generate the 800 nm pump pulse, a fraction of the 800 nm beam was directed through a spectral parametric amplifier (OPera Solo). The probe beam was derived from another fraction of the 800 nm pulse, which was focused onto a Ti: sapphire crystal. The probe beam was sent through a motorized delay stage, allowing temporal control of the probe arrival time relative to the pump pulse. The maximum time decay achievable was approximately 7 ns, determined by the physical length of the delay line. After temporal adjustment, the 800 nm probe pulse was focused into a Sapphire crystal, where it underwent self-phase modulation to generate a white-light continuum. The spectral coverage of this continuum depended on the crystal thickness; a thinner crystal provided a visible range of 450-750 nm. The generated continuum was split by a beam splitter into two paths: one directed through the sample (probe) and the other used as a reference to correct for the pulse-to-pulse fluctuations and enhanced the signal-to-noise ratio. Both beams were coupled into separate multichannel spectrometers (equipped with Si or InGaAs detectors) via optical fibres. The probe beam was spatially ad temporally overlapped with the pump beam at the sample position. Transient absorption signals(∆OD) were determined by the difference in absorbance between the excited and the ground states. This was achieved by modulating the pump beam using an optical chopper operating at 500 Hz, effectively blocking every alternate pump pulse. The pulse energies were measured using an energy meter (Thorlab, PM400, S120VC).

Before measurements, FeCuO_x_ and CuO_x_ films were pre-treated under a 10%H_2_/Ar flow at 513 K for 40 minutes to ensure complete reduction of Cu^2+^ to Cu^0^. After this activation, the films were denoted as “Cu/Fe_2_O_3_” and “Cu” to reflect their chemical nature.

**Online mass spectra monitoring experiment**

A 20 mg catalyst was deposited on a glass fibre membrane using the same method described above. The membrane was sealed in a reactor with a light window (diameter 4.5 cm), and the reactor outlet was connected to a mass spectrometer (HAL RC RGA201). The reactor and gas line were purged with Ar at 150 mL min^-1^ for 0.5 h to remove residual gases. The Ar flow rate was then reduced to 50 mL min^-1^, and a Xenon lamp (Perfectlight. Co., PLS-SME400EH1) equipped with a 700 nm long-pass filter and irradiance similar to the catalytic experiment was used to illuminate the catalyst for 4 h, until the mass spectrometer signal reached a steady state, ensuring removal of surface adsorbates (e.g., H_2_O). After illumination, the catalyst was cooled to room temperature under Ar flow for an additional 6 h until the baseline stabilised. Subsequently, the 50 mL min^-1^ Ar was redirected through a bubbler containing 20 mL ethanol (pretreated to remove air). This step lasted 0.5 h to achieve adsorption equilibrium and a stable baseline in the mass spectrometer. Once the signal stabilised, a monitoring program was initiated to record signals for selected molecular fragments: m/z = 2 (H_2_), 18 (H_2_O), 43 (CH_3_CHO), and 46 (EtOH). Note: CH_3_CHO was monitored at m/z 43 instead of its molecular weight (44) to avoid overlap with CO_2_. The Xenon lamp was switched on at around 80 s after the start of the recording program.

**NH_3_-TPD experiments**

NH_3_ temperature programmed desorption (NH_3_-TPD) experiments were conducted using an online Nicolet Antaris IGS system to evaluate changes in the number of acid sites on the catalysts. The samples were pretreated with 20% O_2_/N_2_ at 400°C for 30 minutes, then exposed to 500 ppm NH_3_/N_2_ at 50°C until saturation. This was followed by a 1-hour purge with N_2_. Subsequently, the temperature was ramped from 50°C to 700°C at a rate of 10°C/min, and the NH_3_ concentration was continuously monitored.

**EtOH/H_2_O-TPD-MS experiments**

The temperature-programmed desorption (TPD) experiments coupled with mass spectrometry (MS) were performed using a Micromeritics Autochem III chemisorption analyzer equipped with a OmniStar® GSD 350 mass spectrometer. Before the adsorption experiment, the catalyst was first pretreated at 300°C under an Ar atmosphere (100 mL min^-1^) for 30 min, followed by cooling to room temperature. Subsequently, Ar carrier gas saturated with EtOH/H_2_O vapor (molar ratio = 2:1, 50 mL min^-1^) was introduced, and the sample was treated at 240°C for 1 h. After this step, pure Ar gas (100 mL min^-1^) was passed through the system at 300°C for 1 h to remove residual species, and the catalyst was then cooled to room temperature under Ar. Thereafter, Ar carrier gas saturated with EtOH/H_2_O vapor (molar ratio = 2:1, 50 mL min^-1^) was introduced again, and the sample was treated at 240°C for another 1h to ensure adsorption equilibrium on the catalyst surface. Upon completion of the adsorption step, heating was stopped and the system was switched to pure Ar gas (50 mL min^-1^) without EtOH/H_2_O vapor to remove weakly adsorbed EtOH/H_2_O species. Simultaneously, the mass spectrometer was turned on to record the data. Once the baseline of each monitored species became stable, the current temperature was recorded, and TPD was initiated: the sample was heated from the recorded temperature (180°C) to 500°C under Ar flow (50 mL min^-1^) at a heating rate of 5°C min^-1^. The mass spectrometer was set to monitor the following characteristic m/z signals: 18 (H_2_O), 45 and 31 (EtOH), 43 and 29 (CH_3_CHO).

**H_2_-TPR experiments**

A 70 mg sample was pretreated in 5% O_2_/He (30 mL min^-1^) at 673 K for 30 minutes, then cooled to 323 K. Subsequently, argon (10 mL min^-1^) was used to purge the sample to remove the residues. After stabilisation, the TPR was performed by heating the sample to 873 K at a rate of 10 K min^-1^ under a 10% H_2_/Ar flow (30 mL min^-1^). The signal was collected using a TCD detector on a Micromeritics AutoChem II 2920 chemisorption analyser.

**In-situ DRIFTS experiments**

In-situ diffuse reflectance infrared Fourier transform spectroscopy (DRIFTS) measurements were conducted using a Thermo Nicolet iS50 FTIR spectrometer equipped with a Harrick diffuse reflectance accessory. 15 mg of catalyst powder was loaded into the in-situ reaction chamber. The catalyst was first pre-activated by flowing 30 mL min^-1^ of Ar gas bubbled through an ethanol/H_2_O mixture (75 vol% ethanol) at 513 K for 1 hour. (*Note: To closely replicate the actual reaction environment, the temperature of the reactor was set to 523 K, ensuring that the catalyst surface reached approximately 513 K—matching the optimised reaction conditions*). Afterward, the system was cooled to room temperature, and the gas flow was switched to pure Ar to purge the chamber at 300 °C for 1 hour, ensuring the removal of all surface adsorbates. The system was then cooled again to room temperature. Subsequently, Ar bubbled through ethanol/H_2_O (75 vol% ethanol) was introduced for 20 minutes to allow ethanol adsorption on the catalyst surface. IR spectra were recorded every 5 minutes during this period. Following the adsorption equilibrium, the inlet and outlet valves were closed, and the catalyst was rapidly heated to 513 K. The reaction was allowed to proceed under static conditions for 20 minutes, during which IR spectra were also recorded every 5 minutes to monitor the evolution of surface intermediate species at high temperature.

**DFT simulations**

All spin-polarized DFT calculations were performed using the Vienna Ab-initio Simulation Package (VASP).^[4]^ The projector augmented wave (PAW) method^[5]^ and the Perdew-Burke-Ernzerhof (PBE) functional^[6]^ under the generalized gradient approximation (GGA)^[7]^ were applied in the calculations. Van der Waals interactions between adsorbates and the surfaces were described using the DFT-D3 correction as proposed by Grimme.^[8,9]^ The kinetic energy cut-off was set to 400 eV, and the force threshold for structure optimizations was 0.05 eV/Å.

To model the Fe_2_O_3_(0001) surface, we constructed a periodic slab based on bulk lattice parameters (a = b = 5.04 Å, c = 13.72 Å)^[10]^. Given the antiferromagnetic nature of Fe_2_O_3_ and the presence of local magnetic moments on Fe atoms, we adopted the primitive rhombohedral unit cell with the (+ – – +) magnetic configuration (Figure S31a), which has been previously shown to be the most stable arrangement^[11]^. A *p*(2 × 2) slab containing 12 layers of Fe atoms and 6 layers of O_3_ was employed to model single-Fe-terminated Fe_2_O_3_(0001) surfaces. The top nine atomic layers were fully relaxed while the bottom nine atomic layers were fixed to mimic the bulk region. The Cu/Fe_2_O_3_ model was generated by placing a Cu_4_ cluster on the Fe_2_O_3_ (0001) surface, as shown in Figure S31b. A vacuum gap of 20 Å was introduced to prevent spurious interactions between periodic images, and a 2 × 2 × 1 *k*-point mesh was used for Brillouin zone sampling. Due to the strong *d*-electron correlation effects of Fe, all calculations were carried out using the DFT+*U* method with *U* = 4 eV and *J* = 1 eV on Fe-*d* orbital as reported previously^[10,12]^.

The transition states (TSs) of surface reactions were located using a constrained optimization scheme and were verified when (i) all forces on the relaxed atoms vanish and (ii) the total energy is a maximum along the reaction coordinate but a minimum with respect to the rest of the degrees of freedom^[13–15]^.

Figure S1 a. Schematic of initial flow reactor setup, where ethanol and water were introduced via a gas bubbler with Ar carrier gas (MFC is mass flow controller). b. Schematic of the reactor structure.

Note S1: The complete configuration of reactor with detailed dimensions is provided in Figure S1b. The reactor consists of two main parts: a base (diameter: 77 mm) and a cap (diameter: 77.5 mm). The cap includes an optical window (diameter: 30 mm). A rubber O-ring (outer diameter: 77 mm, inner diameter: 60 mm, thickness: 8 mm) is placed between the cap and base to ensure a tight seal. The entire assembly is secured using an attaching clamp to maintain a closed and stable reaction environment.

Figure S2 a. The transmittance spectrum of the 700 nm long-pass filter. b. The light spectrum of the Xenon lamp provided by the bulb manufacturer Excelitas, showing an output range from 200 to 2500 nm).^[16]^

Figure S3 The carbon-containing products of IR-driven acetaldehyde synthesis on Cu/Fe_2_O_3_, Cu/MnO_x_, Cu/NiO_x_ and Cu/CoO_x_ (Reaction condition: 20 mg cat., 30 mL min^-1^ Ar carrier gas, 60 mL EtOH/H_2_O (molar ratio 1:1) in the gas bubbler, 300 W Xe (700 nm<λ<2500 nm), no external heating.)

Figure S4 The H_2_/CH_3_CHO ratio achieved by IR-driven acetaldehyde synthesis on Cu/Fe_2_O_3_, Cu/MnO_x_, Cu/NiO_x_ and Cu/CoO_x_ (Reaction condition: 20 mg cat., 30 mL min^-1^ Ar carrier gas, 60 mL EtOH/H_2_O (molar ratio 1:1) in the gas bubbler, 300 W Xe lamp (700 nm<λ<2500 nm), no external heating.)

Figure S5 The H_2_ yield rate over Cu/Fe_2_O_3_ with different Cu/Fe molar ratios (Reaction condition: 20 mg cat., 30 mL min^-1^ Ar carrier gas, 60 mL EtOH/H_2_O (molar ratio 1:1) in the gas bubbler, 300 W Xe lamp (700 nm<λ<2500 nm), no external heating.)

Table S1 The weight percentage of Fe and Cu in the optimised Cu/Fe_2_O_3_ detected by ICP-OES

| Element | Fe | Cu |
| --- | --- | --- |
| Percentage (wt. %) | 23.59 | 49.18 |

Figure S6 The H_2_ yield rate over Cu/Fe_2_O_3_ using one 700 nm long-pass filter or two 700 nm long-pass filters (Reaction condition: 20 mg cat., 30 mL min^-1^ Ar carrier gas, 60 mL EtOH/H_2_O (molar ratio 1:1) in the gas bubbler, 300 W Xe lamp (700 nm<λ<2500 nm, one filter/two filters), no external heating.)

Note S2: To verify that this process is driven by infrared (IR) light rather than the leakage of ultraviolet-visible (UV-Vis) light from the 700 nm long pass filter, the experiment was conducted as illustrated in Figure S6. If the observed activity was due to residual UV-Vis photons passing through a single filter, the addition of a second filter should significantly diminish the activity. The comparable activity observed in both experiments indicates that the process is primarily driven by IR photons.

Figure S7 Schematic of upgraded reactor setup. The system includes a syringe pump and evaporator to precisely control the concentration of ethanol/water (EtOH/H_2_O) in the argon (Ar) carrier gas flow.

Figure S8 a. Carbon-containing products of the IR-driven acetaldehyde synthesis over Cu/Fe_2_O_3_ under varying irradiances. b. H_2_ yield rate of the IR-driven acetaldehyde synthesis over Cu/Fe_2_O_3_ under varying irradiances. c. H_2_/CH_3_CHO ratios for IR-driven acetaldehyde synthesis over Cu/Fe_2_O_3_ under varying irradiances. (Reaction condition: 60 mL min^-1^ Ar carrier gas, 20 mg cat. for Cu/ Fe_2_O_3_, 300 W Xe (700 nm<λ<2500 nm), room temperature, EtOH/H_2_O (molar ratio: 2:1), syringe pump 0.01 ml min^-1^. The error bars were obtained from three independent reactions.)

Figure S9 a. Carbon-containing products yield rates of the IR-driven acetaldehyde synthesis over Cu/ Fe_2_O_3_ under varying gas hourly space velocities (GHSV). b. H_2_ yield rate of the IR-driven acetaldehyde synthesis over Cu/Fe_2_O_3_ under varying GHSV. c. H_2_/CH_3_CHO ratios for IR-driven acetaldehyde synthesis reaction over Cu/ Fe_2_O_3_ under varying GHSV. d. EtOH conversion and CH_3_CHO selectivity for IR-driven acetaldehyde synthesis reaction over Cu/ Fe_2_O_3_ under varying GHSV. (Note: the GHSV is the ratio of the Ar flow rate to the mass of catalyst; Reaction condition: 30, 60, 120 mL min^-1^ Ar carrier gas, the liquid injection rate of EtOH/H_2_O (molar ratio: 2:1) was set to 0.005, 0.01, and 0.02 ml min^-1^ to maintain the same concentration, 20 mg Cu/ Fe_2_O_3_ catalyst, 300 W Xe (700 nm<λ<2500 nm, 1226 mW cm^-2^).)

Figure S10 a. Effect of varying EtOH/H_2_O molar ratios on the H_2_ yield rate. b. Effect of varying EtOH/H_2_O molar ratios on H_2_/CH_3_CHO ratio. c. Effect of varying EtOH/H_2_O molar ratios on EtOH conversion and CH_3_CHO selectivity. (Reaction condition: 120 mL min^-1^ Ar carrier gas, 20 mg catalyst, 300 W Xe lamp (700 nm<λ<2500 nm, 1226 mW cm^-2^), 0.02 ml min^-1^ liquid injection rate, no external heating. The error bars were obtained from three independent reactions.)

Figure S11 Thermal distribution of Cu/Fe_2_O_3_ under IR irradiation at an irradiance of 1226 mW cm^-2^. The light window diameter (3 cm) is smaller than the catalyst bed diameter (3.6 cm), so only the red and yellow regions correspond to the irradiated area.

Figure S12 Stability evaluation of Cu/Fe_2_O_3_ under the optimised reaction conditions (Reaction conditions: 120 mL min^-1^ Ar carrier gas, EtOH/H_2_O (molar ratio: 2:1), 0.02 ml min^-1^ liquid injection rate, 20 mg cat., 300 W Xe lamp (700 nm < λ < 2500 nm, 1226 mW cm^-2^))

Note S3: Some fluctuations were observed during the experiment due to two unavoidable factors: (1) slight changes in feedstock concentration when refilling the syringe pump over extended testing periods; and (2) the switch from a small syringe pump to a larger to reduce refill frequency, which introduced additional fluctuations in feed rates. The production rates of both H_2_ and CH_3_CHO exhibit an initial decline during the first ~70 hours, followed by a slower decease between 70-140 hours, thereafter, the activity stabilises at approximately 30 mmol g^-1^ h^-1^, which is an order of magnitude higher than most reported IR-driven photocatalysts.

Figure S13 Carbon balance during the stability evaluation of Cu/Fe_2_O_3_ under the optimised reaction conditions. (Reaction conditions: 120 mL min^-1^ Ar carrier gas, 1226 mW cm^-2^ irradiance, 0.02 mL min^-1^ liquid injection rate, EtOH/H_2_O molar ratio = 2:1. 20 mg catalyst and 300 W Xe lamp with a 700 nm long-pass filter (700 nm < λ <2500 nm).)

Table S2 Performance of Cu/Fe_2_O_3_ and other representative IR-driven catalysts

| Catalysts | Reaction conditions | Initial production (mmol g^-1^_cat_ h^-1^) | Selectivity to valuable oxidation products | Initial TOF_w_  (mmol g_M_^-1^ h^-1^) | TON_w_  (mmol g_M_^-1^) |
| --- | --- | --- | --- | --- | --- |
| Cu/Fe_2_O_3_ (This work) | 20 mg cat., 120 mL min^-1^ Ar carrier gas, 0.02 ml min^-1^, EtOH/H_2_O (molar ratio: 2:1), 300 W Xe (700 nm< λ <2500 nm, 1226 mW cm^-2^), no external heating, catalyst surface 240°C. | H_2_: 232;  CH_3_CHO: 237 | CH_3_CHO: 97.7% | H_2_: 472  CH_3_CHO: 482 | H_2_: 27051  CH_3_CHO: 29937 |
| Black P NS/Pt/TiO_2_^[17]^ | 2 mg cat., 5 mL methanol/H_2_O (v/v, 1/4), 320 W Xe (λ>780 nm, 200 mW cm^-2^). | H_2_: 0.205 | - | 6.8 | 81.6 |
| Au cube/cage-TiO_2_-Pd Cube^[18]^ | 15 mg cat., 20 mL methanol/H_2_O(v/v, 1/3), 300 W Xenon lamp (λ>780 nm, 18 mW cm^-2^). | H_2_: 0.00028 | - | 0.096 | 1.5 |
| Pt/CuS/CdS^[19]^ | 30 mg cat., 100 mL methanol/H_2_O (v/v, 1/4), 300 W Xenon lamp (800 nm< λ <2500 nm, 50 mW cm^-2^). | H_2_: 0.016 | - | 0.59 | 2.3 |
| Black P/Pt/RGO^[20]^ | 0.2 mg Black P, RGO 0.1 mg, Pt 75 μg, 3.8 mL H_2_O, 200 μL EDTA solution (0.5 M), 320 W Xenon lamp (780 nm< λ <1800 nm, 405 mW cm^-2^). | H_2_: 0.84 | - | 3.4 | 13.6 |
| WS_2_/black phosphorous^[21]^ | 0.2 mL BP/WS_2_, 0.2 mL EDTA solution (0.5 M), 4.6 mL H_2_O, 320 W Xenon lamp (780 nm< λ <1800 nm, 400 mW cm^-2^). | H_2_: 3.77 | - | 37.7 | 490.1 |
| TiO_2-x_^[22]^ | 100 mg cat., 100 mL methanol/H_2_O (v/v, 1/4), 750 W Xenon lamp (λ>750 nm). | H_2_: 0.0325 | - | 0.00325 | 0.1625 |
| TiO_2_/Ag_2_O^[23]^ | 12.5 mg cat., 20 mL methanol/H_2_O (v/v, 1/9), IR emitting diodes (850 and 950 nm). | H_2_: 1.144 | - | 4.1 | - |
| Black P/g-C_3_N_4_^[24]^ | 1.5 mg cat., 5 mL methanol/H_2_O (v/v, 1/4), 320 W Xenon lamp (780 nm< λ <1800 nm, 300 mW cm^-2^). | H_2_: 0.101 | - | 0.505 | 13.13 |
| Black P/Au/La_2_Ti_2_O_7_^[25]^ | 1.5 mg cat., 5 mL methanol/H_2_O (v/v, 1/4), 320 W Xenon lamp (780 nm< λ <1800 nm, 300 mW cm^-2^). | H_2_: 0.3 | - | 10 | 30 |
| β-NaYF_4_:Yb^3+^, Tm^3+^@Ag_3_PO_4_@Black P^[26]^ | 15 mg cat., 100 mL glycerol/H_2_O (v/v, 1/1), 10 W laser light (980 nm). | H_2_: 0.146 | - | 0.73 | 14.6 |

**Note:*

*1. Since no IR-driven catalysts using ethanol as the oxidant have been reported, data from systems using methanol, EDTA, etc. were used for this comparison.*

*2. H_2_ production was used to calculate TOF_w_ and TON_w_ value (normalised to the weight of the active component for H_2_ production), as existing studies report only H_2_ yields.*

*3. TON_w_ for this work was calculated from the H_2_ production and CH_3_CHO production measured in the long-term stability test, respectively.*

*4. Calculation details were provided in the Method section.*

Table S3 Performance of Cu/Fe_2_O_3_ and representative UV–Vis-Driven photocatalysts and thermocatalysts for ethanol conversion under neutral conditions

| Catalysts | Reaction conditions | Initial production  (mmol g^-1^_cat_ h^-1^) | Selectivity to valuable oxidation products | Initial TOF_w_  (mmol g_M_^-1^ h^-1^) | TON_w_  (mmol g_M_^-1^) | Initial TOF_a_ (h^-1^) | TON_a_ |
| --- | --- | --- | --- | --- | --- | --- | --- |
| Cu/Fe_2_O_3_ (This work) | 300 W Xe (700 nm< λ <2500 nm, 1226 mW cm^-2^), 120 mL min^-1^ Ar carrier gas, 0.02 ml min^-1^, EtOH/H_2_O (molar ratio: 2:1), 20 mg catalyst, no external heating, catalyst surface 240°C. | H_2_: 232; CH_3_CHO: 237 | CH_3_CHO: 97.7% | H_2_: 472  CH_3_CHO: 482 | H_2_: 27051  CH_3_CHO: 29937 | H_2_: 363  CH_3_CHO: 370 | H_2_: 20787  CH_3_CHO: 23005 |
| CoTiO_3_/TiO_2_^[27]^ (UV catalyst) | 365±5 nm UV LEDs (79.1 mW cm^-2^), 10 vol% EtOH solution, 2.0 mg catalyst. | H_2_: 3.8; | - | 3.84 | 403 |  |  |
| Ni/ montmorillonite/TiO_2_^[28]^ (UV-Vis catalyst) | 200 W Hg lamp (150 mW cm^-2^), 80% EtOH solution, 0.25 g catalyst. | H_2_: 3.47; CH_4_: 0.019; C_2_H_4_: 0.00195; C_3_H_6_: 0.0189. | CH_4_: 23.9%; C_2_H_4_: 4.9%; C_3_H_6_: 71.2%. | 116 | 2784 |  |  |
| Au/ZnO^[29]^ (UV-Vis catalyst) | 300 W Hg-Xe lamp (λ>300 nm), 8 mL 20 vol% EtOH solution, 7 mg catalyst. | H_2_: 0.427; CH_4_: ~0.335; CO: ~0.143 | CH_4_: 70.1%; CO: 29.9% | 147 | - |  |  |
| CuS/TiO_2_^[30]^ (UV-Vis catalyst) | 300 W Xe lamp (1000 mW/cm^2^), 5 mL EtOH solution, 0.313 mg-catalyst/cm^2^. | H_2_: 51.61; CH_3_CHO: 48.63. | CH_3_CHO: 97% | 403 | - |  |  |
| NiCu/SiO_2_^[31]^ (Visible catalyst) | Xe lamp (AM1.5, 574 mW cm^-2^), 50 mL min^-1^ Ar, 6.5% ethanol, 10 mg catalyst, catalyst surface 209°C. | H_2_: 176.6; | - | 1598 | 7990 |  |  |
| TiO_2_/FePS_3_^[32]^ (UV-Vis catalyst) | 350 W Xe lamp (λ>350 nm), 80 mL 10 vol% EtOH solution, 20 mg catalyst, no external heating. | H_2_: 0.0995 | - | 2.4 | 21.6 |  |  |
| Pt/TiO_2_^[33]^ (UV catalyst) | 300 W Hg lamp (λ=365 mm), 200 mL 30 vol% EtOH solution, 1.0 g catalyst. | H_2_: 8.5; 2,3-Butanediol: 4 | 2,3-Butanediol: 90% | 850 | 51000 |  |  |
| W_18_O_49_^[34]^ (Visible-NIR catalyst) | 300 W Xe lamp (420-1000 nm), 50 μL EtOH, 0.2 g catalyst, catalyst surface 300°C. | C_2_H_4_:~24 | C_2_H_4_:~100% | C_2_H_4_:~24 | ~24 |  |  |
| Au/TiO_2_^[35]^ (UV catalyst) | 125 W Hg lamp (UV-rich), 1.4 bar Ar, 25 mL EtOH, 25 mg catalyst. | H_2_: 29.677; CH_3_CHO: 29.047. | CH_3_CHO: ~99.5% | 1746 | 3492 |  |  |
| Cu/SiO_2_^[36]^ (Thermocatalyst) | 0.1 g cat., 30 mL/min He, 200 µL/h ethanol, GHSV = 34930 mL g^-1^ h^-1^, 553 K | CH_3_CHO: 30.9 | CH_3_CHO: ~99% | - | - | 51.3 | 25650 |
| NiCu^[37]^ (Thermocatalyst) | 0.03 g cat., 7.5 mL min^-1^ He, 5.6% ethanol, 523 K | CH_3_CHO: 9.1 | CH_3_CHO: ~100% | - | - | 7.2 | 309.6 |
| Cu/ZrO_2_^[3]^ (Thermocatalyst) | 0.2 g cat., 0.00526 g min^-1^ EtOH, P_EtOH_ = 7.0 kPa, 573 K | CH_3_CHO: 10.6 | CH_3_CHO: 54.1% | - | - | 208.8 | 2505.6 |
| CuO-Cr_2_O_3_/Al_2_O_3_^[38]^ (Thermocatalyst) | 1.578 g cat., 2 mL h^-1^ ethanol, WHSV = 1 h^-1^, 573 K | CH_3_CHO: ~8.4 | CH_3_CHO: 70.4% | - | -- | 22.32 | 334.8 |
| Cu/ZrO_2_^[39]^ (Thermocatalyst) | 0.5 g cat., 0.026 g min^-1^ ethanol, 10 mL min^-1^ He, 473 K | CH_3_CHO: 3.97 | CH_3_CHO: 21% | - | - | 14.8 | 369 |
| Cu-MFI^[40]^ (Thermocatalyst) | 0.2 g cat., WHSV = 0.53 h^-1^, 523 K | CH_3_CHO: 10.4 | CH_3_CHO: 93% | - | - | 14 | 7020 |

**Note: 1. UV-Vis catalysts: H_2_ was used as the performance indicator because it is more frequently reported than carbon-containing products, and the types of carbon-containing products varied across different systems. As most UV-Vis catalysts reported only the weight of active component, TOF_w_ and TON_w_ (based on the weight of active component) were calculated for comparison.*

*2. Thermocatalysts: Ethanol dehydrogenation to CH_3_CHO reaction was used for this comparison and CH_3_CHO was used as the performance indicator. When multiple optimised results were reported, the one with the highest CH_3_CHO selectivity was selected. Because most thermocatalysts reported the number of surface active sites, TOF_a_ and TON_a_ (based on the number of surface active sties) were used.*

*3. TON for this work was calculated from the H_2_ production and CH_3_CHO production measured in the long-term stability test, respectively.*

*4. Calculation details were provided in the Method section.*

Figure S14 TON_w_ and initial TOF_w_ of Cu/Fe_2_O_3_ and representative UV-Vis-driven catalysts for ethanol conversion under neutral conditions (1: CoTiO_3_/TiO_2_^[27]^; 2: Ni/montmorillonite/TiO_2_^[28]^; 3: Au/ZnO^[29]^; 4: CuS/TiO_2_^[30]^; 5: NiCu/SiO_2_^[31]^; 6: TiO_2_/FePS_3_^[32]^; 7: Pt/TiO_2_^[33]^; 8: W_18_O_49_^[34]^; 9: Au/TiO_2_^[35]^. *Note*: No reaction-time information was reported for catalyst 3 and 4, so TON could not be determined. Catalyst 8 reported only C_2_H_4_ formation (no H_2_), and therefore TOF_w_ and TON_w_ were calculated using C_2_H_4_ production. TON_w_ and initial TOF_w_ were calculated based on the weight of the active component for H_2_ production because surface active sites are generally not reported. Detailed reaction conditions can be found in Table S3.

Note S4: Our catalyst exhibits high selectivity toward CH_3_CHO (97.7%), whereas UV-Vis photocatalytic systems often produce different oxidation products. Therefore, H_2_ production is again used as the common indicator for the comparison. Our catalyst is at the advanced level of initial TOF_w_. Two catalysts (5 and 9) show higher initial TOF_w_, but both are surpassed by our catalyst when evaluated using TON_w_, which accounts for the durability. Only one catalyst (7) exceeds our catalyst in both TOF_w_ and TON_w_; however, it relies on expensive noble metal Pt and an extremely strong Hg lamp. The TON_w_ ranking remains unchanged even under rough irradiance normalisation. Photon-driven systems typically rely on high-energy photons to generate energetic charge carriers. This becomes progressively more difficult at longer wavelengths, as reflected in the pronounced gap between IR- and UV-Vis-driven systems (Figure 2c and Figure S14). In contrast, our catalyst harnesses low-energy IR photons (up to 2500 nm) to activate a photon-to-phonon pathway, bypassing the constraints of bandgap excitation and redox potentials. The high performance of our catalyst underscores the promises of this alternative excitation mechanisms.

Figure S15 TON_a_ and initial TOF_a_ of Cu/Fe_2_O_3_ and representative thermocatalysts for ethanol dehydrogenation to acetaldehyde (1: Cu/SiO_2_^[36]^; 2. NiCu^[37]^; 3. Cu/ZrO_2_^[3]^; 4. CuO-Cr_2_O_3_/Al_2_O_3_^[38]^; 5. Cu/ZrO_2_^[39]^; 6. Cu-MFI^[40]^). TON_a_ and initial TOF_a_ were calculated based on the number of surface active sites for CH_3_CHO production. Detailed reaction conditions can be found in Table S3.

Note S5: One thermocatalyst (catalyst 1) shows a slightly higher TON_a_ than this work, indicating superior long-term stability considering its seven-fold lower initial TOF_a_. This highlights the future research of our work is to enhance the interaction between the substrate and Cu so that the durability of our catalyst would be improved.

Figure S16 Thermal distribution of Cu/Fe_2_O_3_ under electronic heating at a set temperature of 200 °C.

Figure S17 Thermal distribution of Cu/Fe_2_O_3_ under IR irradiation at an irradiance of 405 mW cm^-2^.

Note S6: Since the maximum setting temperature of the reactor under thermal heating is limited to 200°C, we evaluated the catalytic activity at this temperature, which yields about 198 µmol h^-1^ for CH_3_CHO. Then, we used a thermal camera to analyse the thermal field distribution and estimated the heat input more accurately at this time. As shown in Figure S16, the catalyst exhibits two distinct regions: a low-temperature centre (143-157°C) and a high-temperature edge (163-181°C), attributed to the edge’s proximity to the stainless-steel ring, which facilitates heat conduction from the reactor. The total thermal heat input for a one-hour reaction is expressed as Q_t_ = Q_1_ + Q_2_, where Q_1_ is the energy absorbed by the reaction, and Q_2_ is the energy needed to maintain the working temperature. Radiation heat loss is neglected in this calculation because the reaction temperature is relatively low, making its contribution insignificant.

Calculation of Q_1_:

$Q_{1}=\Delta H^{^{\circ}}\times y\times t=68.91 kJ {mol}^{-1}\times198 \mu mol h^{-1}\times1 h=13.6 J$

Calculation of Q_2_:

$$S_{1}=\pi r_{1}^{2}=3.14\times{1.35}^{2}{cm}^{2}=5.72 {cm}^{2}$$

$S_{2}=\pi r_{2}^{2}-\pi r_{1}^{2}=3.14\times{1.8}^{2}{cm}^{2}-3.14\times{1.35}^{2}{cm}^{2}=4.45 {cm}^{2}$

$Q_{c}=K\times S_{1}\times\left( T_{L}-T_{0} \right)\times t+K\times S_{2}\times\left( T_{H}-T_{0} \right)\times t=50\frac{W}{m^{2}\cdot℃}\times5.72 {cm}^{2}\times\left( 150^{\circ}C-25^{\circ}C \right)\times1 h+50\frac{W}{m^{2}\cdot℃}\times4.45 {cm}^{2}\times\left( 172^{\circ}C-25^{\circ}C \right)\times1 h=24645 J$

$$n_{Ar}=\frac{V_{Ar}}{V_{m}}=\frac{120 mL {min}^{-1}}{24.5 L {mol}^{-1}}=8.16\times{10}^{-5} mol s^{-1}$$

$Q_{g}=n_{Ar}\times c_{Ar}\times\left( T_{L}-T_{0} \right)\times t\times\frac{S_{1}}{S_{1}+S_{2}}+n_{Ar}\times c_{Ar}\times\left( T_{H}-T_{0} \right)\times t\times\frac{S_{2}}{S_{1}+S_{2}}=8.16\times{10}^{-5} mol s^{-1}\times20.79 \frac{J}{mol\cdot^{\circ}C}\times\left( 150^{\circ}C-25^{\circ}C \right)\times1 h\times\frac{{5.72 cm}^{2}}{5.72 {cm}^{2} + {4.45 cm}^{2}}+8.16\times{10}^{-5} mol s^{-1}\times20.79 \frac{J}{mol\cdot^{\circ}C}\times\left( 172^{\circ}C-25^{\circ}C \right)\times1 h\times\frac{{4.45 cm}^{2}}{5.72 {cm}^{2} + {4.45 cm}^{2}}=822 J$

$$Q_{2}= Q_{c}+Q_{g}=24645 J+822 J=25467 J$$

Calculation of Q_t_:

$$Q_{t}= Q_{1}+Q_{2}=13.6 J+25467 J=25.5 kJ$$

Definitions:

- ∆H° = 68.91 kJ mol^-1^: Enthalpy change of the reaction (C_2_H_5_OH (g) → CH_3_CHO (g) + H_2_ (g));
- y = 198 µmol h^-1^: CH_3_CHO production rate;
- t = 1 h: Reaction time.
- S_1_, S_2_: Area of low- and high-temperature regions;
- r_1_ = 1.35 cm: Radii of the low-temperature region;
- r_2_ = 1.8 cm: Radii of the whole region;
- T_L_ = 150°C, T_H_ = 172°C: Representative temperature of the low- and high-temperature region, corresponding to the midpoint of 143-157°C and 163-181°C, respectively;
- T_0_ = 25°C: Room temperature used as the baseline for heat calculations;
- Q_c_: Heat required to compensate for heat dissipation via forced convection by carrier gas flow in both low- and high-temperature regions;
- K = 50 W m^-2^ °C^-1^: Empirical forced convective heat transfer coefficient used to evaluate heat loss from the catalyst surface to the gas flow;
- n_Ar_: Molar flow rate of carrier gas (Ar) during the reaction;
- V_m_ = 24.5 L mol^-1^: Molar volume of gas taken at 25°C;
- V_Ar_: Volumetric flow rate of carrier gas (Ar) during the reaction;
- Q_g_: Heat required to raise the carrier gas from room temperature (T_0_) to the respective working temperatures (T_L_ and T_H_) in both low- and high-temperature regions (assuming homogenous distribution over the catalyst surface and neglecting minor fractions of ethanol and water vapor);
- c_Ar_ = 20.79 J mol^-1^ °C^-1^: Specific heat capacity of Ar.

Therefore, thermal heat input is estimated as 25.5 kJ. Normalising energy consumption to CH_3_CHO production (198 µmol) gives a thermal energy requirement of 129 kJ per mmol of CH_3_CHO.

To enable a fair comparison with IR-driven system, we adjusted the IR irradiance to achieve a CH_3_CHO production rate comparable to that under thermal heating. At an IR irradiance of 405 mW cm^-2^, the CH_3_CHO production rate reaches 164 µmol h^-1^, slightly lower than the 198 µmol h^-1^ observed under thermal heating. A thermal camera was also used to analyse the thermal field distribution (Figure S17). Notably, the light window diameter (3 cm) is smaller than the catalyst bed diameter (3.6 cm), so only the red and yellow regions correspond to the irradiated area. Interestingly, the thermal distribution trend under IR irradiation shows the opposite trend to that under electric thermal heating: temperatures decrease from the centre (ca. 125-148°C) toward the edge (ca. 100-125°C). This difference arises from the distinct heating mechanisms, thermal conduction from the stainless-steel bottom and edge ring in electric heating versus central irradiation with heat loss to the surroundings under IR light. The slightly lower overall temperature range under IR irradiation (405 mW cm^-2^) explains the marginally lower CH_3_CHO production rate.

The energy input under IR irradiation (Q_L_) is calculated as:

$$Q_{L}=I\times S_{L}\times t=405 mW {cm}^{-2}\times7 {cm}^{2}\times1 h=10.2 kJ$$

Definitions:

- I = 405 mW cm^-2^ : IR irradiance adjusted to achieve a CH_3_CHO production rate comparable to thermal heating;
- S_L_ = 7 cm^2^: IR irradiation area;
- t = 1 h: Reaction time.

Thus, the energy input of IR light for a one-hour reaction is 10.2 kJ. With a CH_3_CHO production rate of 164 µmol h^-1^, the IR energy required to produce 1 mmol of CH_3_CHO is calculated as 62 kJ.

Overall, the energy efficiency of thermal heating and IR irradiation can be compared under similar conditions: 129 kJ/mmol_CH3CHO_ (thermal heating) versus 62 kJ/mmol_CH3CHO_ (IR irradiation). In other words, producing the same amount of CH_3_CHO via thermal heating consumes more than twice the energy required by the IR-driven system. It should be noted that the thermal heating value represents the minimum heat required for the reaction, excluding unavoidable heat losses to reactor components such as support membranes and stainless-steel structures. This further highlights that IR-driven ethanol conversion is more energy-efficient than conventional thermal heating, owing to the localised heating effect of IR light, which directly and selectively heats IR-sensitive active sites. However, we acknowledge that this comparison is approximate and empirical. The energy efficiency of thermocatalytic systems could potentially be improved through optimised reactor design and advanced materials, which are beyond the scope of this study. Overall, our results show that IR-driven catalysis offers a promising, clean, and efficient alternative to traditional fossil-fuel-based thermal energy sources for ethanol conversion.

Figure S18 a. The STEM image of Cu/Fe_2_O_3_ before the reaction; b. The lattice fringe analysis of Cu/Fe_2_O_3_ before the reaction; c-g. HAADF-STEM image and EDX elements mapping of Cu/Fe_2_O_3_ before the reaction.

Figure S19 a. The STEM image of Cu/Fe_2_O­_3_ after one hour reaction under optimised reaction conditions. b-e. The EDX elements mapping of Cu/Fe_2_O­_3_ after one hour reaction under optimised reaction conditions.

Figure S20 a. HAADF-STEM images of Cu/Fe_2_O_3_ after reaction. b. EDX element mapping of Figure a. c. Lattice fringe analysis of Fe_2_O_3_. d. Particle size distribution of Fe_2_O_3_. (*Note: The fitting curve was generated by the Gauss model*.)

Figure S21 a. Mass spectral responses for water (m/z = 18), CH_3_CHO (m/z = 43), and H_2_ (m/z = 2) after IR irradiation was turned on under ethanol vapor purge in argon over Cu/Fe_2_O_3_. b. H_2_-TPR profiles of Cu/Fe_2_O_3_.

Note S7: Previous studies have reported that copper oxide can be reduced to metallic copper by ethanol;^[41,42]^ in other words, lattice oxygen in copper oxide can react with ethanol to produce acetaldehyde and water (Equation 1). This process is highly likely to drive the initial reduction of Cu^2+^ species during the reaction.

CuO + CH_3_CH_2_OH → Cu + CH_3_CHO + H_2_O	(Equation 1)

To verify this hypothesis, we conducted an experiment under pure ethanol vapor with Ar flow, using online mass spectrometry (MS) to monitor the initial reaction, as shown in Figure S21a. Notably, after adsorption equilibrium, once IR light was switched on, an increase in the water signal (m/z = 18) is observed, confirming that CuO species can be reduced by ethanol at the initial stage. Simultaneously, signals for CH_3_CHO (m/z =43, chosen instead of molecular weight at 44 to avoid overlap with CO_2_) and H_2_ (m/z = 2) also increases. This indicates that once metallic Cu forms, even in small amounts, they can catalyse ethanol dehydrogenation (Equation 2).

CH_3_CH_2_OH → CH_3_CHO + H_2_	(Equation 2)

To examine whether the H_2_ can reduce CuO to metallic Cu (Equation 3), a H_2_-TPR experiment was conducted, as shown in Figure S21b.

CuO + H_2_ → Cu + H_2_O	(Equation 3)

A distinct peak in the range of ~360-500 K, centred at 460 K, corresponds to the reduction of Cu species in the catalyst.^[43,44]^ In contrast, the broader peak spanning ~540-820 K is attributed to the reduction of Fe species^[45]^. These results indicate that the surface temperature during the reaction is sufficient to drive the reduction of Cu species with the as formed H_2_ via Equation 3.

As shown in Figure S21a, H_2_O signal reaches its maximum after ~180 s light-on and return to the baseline after ~720 s light-on, indicating that oxidised copper species are fully reduced within ~10 min, a timescale consistent with our in-situ XRD and in-situ XPS observations. The H_2_ signal increases gradually and stabilises after around 720 s light-on, coinciding with the cessation of H_2_O production. Careful analysis of the H_2_O curve before its peak reveals two kinetic regions: a steeper slope with 20-70 s light-on, and a moderate slope with 70-180 s light-on. This suggests that direct CuO reduction by ethanol (Equation 1) likely dominates initially, while after sufficient metallic Cu forms, the pathway shifts toward H_2_-mediated reduction (Equation 3). The point at which the H_2_O signal reaches its peak intensity indicates that the majority of CuO species have been reduced, while the remaining oxidised copper species continue to undergo gradual reduction. The H_2_ signal exhibits a consistent trend, with its increase rate slowing after this point, which correlates well with the reduced rate of Cu formation. This alignment between the H_2_ and H_2_O profiles strongly supports the transition from rapid initial reduction to a slower, residual reduction phase. Overall, these online MS results, combined with previous H_2_-TPR data, clearly explain the driving force behind Cu^2+^ reduction during the reaction.

Figure S22 a. The HAADF-STEM image and EDX elements mapping of Cu/Fe_2_O_3_ after 18 hours reaction under optimised reaction conditions. b. The particle size distribution of Cu nanoparticles for Cu/Fe_2_O_3_ after 18 hours reaction under optimised reaction conditions. (*Note: The fitting curve was generated by the Gauss model*.)

Figure S23 The UV-VIS-IR DRS spectrum of Cu/Fe_2_O_3_ before and after reaction.

Note S8: Before reaction, Cu/Fe_2_O_3_ shows a very wide light response up to 2500 nm. In other words, this catalyst has strong light absorption in the IR region. A new, enhanced “light absorption” feature with a relatively flat profile appears after reactions, in particular at the range of ~1000-2500 nm. This is exactly attributed to scattering effect caused by closely packed large Cu nanoparticles in this study.^[46]^ Careful inspection of the spectra also reveal the emergence of a new small peak around 560 nm in the Cu/Fe_2_O_3_ after the reaction, corresponding to the SPR effect of metallic Cu nanoparticles.^[47,48]^ These observations consistent with our structural analysis (STEM, XPS, XRD), which confirms the formation of metallic Cu nanoparticles with an average size of 40 nm during the reaction.

Figure S24 a. ns TAS spectra of FeO_x_ under an argon atmosphere. b. ps-ns TAS spectra of FeO_x_ under an argon atmosphere with methanol as the hole scavenger. c. fs-ps TAS spectra of Cu under an argon atmosphere. (Excitation wavelength: 800 nm. Excitation energy: 0.66 mJ cm^-2^ for FeO_x_ and Cu; 1.1 mJ cm^-2^ for Cu/Fe_2_O_3_)

Note S9: Regarding the possibility of direct intrinsic phonon excitation, a photon can excite a phonon only when their energies match. Infrared-active photons in inorganic solids typically lie in the mid-IR to far-IR region, generally below ~0.2 eV^[49–52]^. Even the highest-energy optical phonons rarely exceed this limit. For example, Fe_2_O_3_ used in our study, the main phonon modes fall within 0.03-0.10 eV, well below the energies of the photons used in our experiments^[53,54]^. Our excitation wavelengths (700-2500 nm, near-IR) correspond to 0.5-1.8 eV, which is far above any phonon mode in Fe_2_O_3_. Consequently, direct photon-phonon absorption is energetically forbidden in our system.

Furthermore, we have considered the possible contribution of direct IR absorption by the substrate molecules under our irradiation range (700-2500 nm). In the gas flow, the three components are Ar, ethanol, and water. Because ethanol and water constitute only a small molar fraction (<10% in total) relative to the large Ar flow, and because their primary IR absorption bands lie mainly in the mid-IR region (4000-400 cm^-1^, 2500-25000 nm) according to the NIST Chemistry WebBook, their direct contributions to photothermal heating can be neglected. As for Ar, its IR absorption arises predominantly from atomic electronic transitions, which occur mainly in the IR region (2000-800 cm^-1^, 5000-12500 nm)^[55]^, also outside the wavelength range of our light source. Therefore, direct IR absorption by gas-phase species, is negligible under our experimental conditions.

Overall, the phonons in our system arise primarily from indirect processes via the recombination of charge carriers, which generates localised thermal fields. We also acknowledge that excited electrons and holes can relax non-radiatively and dissipate part of their energy into the lattice as phonons before recombination.

Figure S25 Schematic of the modified Harrick Praying Mantis reaction cell.

Figure S26 The comparison of H_2_ yield rate under IR light irradiation and by thermal heating (403 K) under dark condition. (Reaction condition: 30 mL min^-1^ Ar carrier gas, 300 W Xe (700 nm<λ<2500 nm), 60 mL EtOH/H_2_O (1:1) in the gas bubbler)

Figure S27 a. The XPS spectra of Fe 2*p* on FeO­_x_ before and after the reaction (the photothermal investigation reaction shown in Figure 4f). b. The XPS spectra of Cu 2*p* on CuO­_x_ before and after the reaction (the photothermal investigation reaction shown in Figure 4f).

Figure S28 a. The control experiment over Cu/Fe_2_O_3_, CuO_x_ and physical mixture FeO_x_+CuO_x_. b. The zoom-in of the rectangle region in Figure S28a. (Reaction condition: 30 mL min^-1^ Ar carrier gas, 20 mg cat. for Cu/Fe_2_O_3_ and 50 mg cat. For CuO_x_, 300 W Xe (700 nm<λ<2500 nm, 480 mW cm^-2^), room temperature, 60 mL EtOH/H_2_O (molar ratio 1:1) in the gas bubbler, no external heating).

Note S10: The physical mixture of FeO_x_ and CuO_x_ exhibits a significantly higher H_2_ yield rate than pure CuO_x_ over an 8-hour test period. However, the H_2_ yield rate of the FeO_x_ and CuO_x_ mixture is approximately 50 times lower than that of Cu/Fe_2_O_3_. This result indicates that the formation of a Cu/Fe_2_O_3_ junction during the reaction is more effective in exerting the synergistic effect.

Figure S29 NH_3_-TPD results of Cu/Fe_2_O_3_ and CuO_x_

Figure S30 a. EtOH/H_2_O-TPD-MS spectra of Cu/Fe_2_O_3_ and CuO_x_. b. Enlarged view of the panel a showing the MS signal at m/z = 43 and 45 for Cu/Fe_2_O_3_ and CuO_x_. c. Enlarged view of panel a showing the MS signal of m/z = 31 over Cu/Fe_2_O_3_ and CuO_x_. d. Enlarged view of panel a showing the MS signal of m/z = 29 over Cu/Fe_2_O_3_ and CuO_x_.

Note S11: In this experiment, adsorption was carried out under Ar saturated with EtOH/H_2_O vapour (molar ratio = 2:1) at 240°C to mimic the reaction atmosphere. During the subsequent desorption, the signals corresponding to H_2_O, EtOH, and CH_3_CHO were monitored, as ethanol can undergo dehydrogenation to CH_3_CHO at high temperatures and the desorbed CH_3_CHO must therefore also be quantified. It should be noted that because the adsorption was performed at a relatively high temperature (240°C), the same as the optimal reaction condition, most weakly bound species would desorb during the gradual cooling process under pure Ar flow. Consequently, the following TPD-MS profiles predominantly reflect the strongly adsorbed surface species, which require relatively higher temperatures to desorb and therefore more reliably indicate the catalysts’ adsorption capability.

Figure S31 Calculated structures (side view) of the Fe_2_O_3_(0001) (a) and Cu_4_/Fe_2_O_3_(0001) surface (b), the spin charge density differences (yellow and blue, the iso-surfaces plotted at the value of 0.05 e/Å^3^) are also shown.

Note S12: To investigate the electronic interaction between Cu and Fe_2_O_3_, Bader charge analysis was performed. The results indicate that the Cu_4_ cluster is only slightly oxidised by the Fe_2_O_3_ support, with an average oxidation state of approximately +0.1 |e|. This minimal charge transfer suggests that Cu remains essentially metallic and experiences negligible electronic perturbation from the oxide. Therefore, the interfacial synergy does not originate from strong electronic modulation of Cu by Fe_2_O_3_, instead, it is primarily arising from a kinetic promotion effect as discussed in the manuscript.

Figure S32 The corresponding mass spectra of CH_3_CHO when using different isotope feedstocks. (Reaction conditions: 20 mg Cu/Fe_2_O_3_ catalyst, 60 mL min^-1^ Ar carrier gas, 300 W Xe lamp (700 nm<λ<2500 nm 1226 mW cm^-2^), ethanol/water (molar ratio: 2:1), 0.01 mL min^-1^ liquid injection rate, no external heating.)

References:

[1]	X. Jie, W. Li, D. Slocombe, Y. Gao, I. Banerjee, S. Gonzalez-Cortes, B. Yao, H. AlMegren, S. Alshihri, J. Dilworth, J. Thomas, T. Xiao, P. Edwards, “Microwave-initiated catalytic deconstruction of plastic waste into hydrogen and high-value carbons” *Nat. Catal.* **2020**, *3*, 902–912.

[2]	S. Sato, R. Takahashi, T. Sodesawa, K. Yuma, Y. Obata, “Distinction between Surface and Bulk Oxidation of Cu through N_2_O Decomposition” *J. Catal.* **2000**, *196*, 195–199.

[3]	A. G. Sato, D. P. Volanti, D. M. Meira, S. Damyanova, E. Longo, J. M. C. Bueno, “Effect of the ZrO2 phase on the structure and behavior of supported Cu catalysts for ethanol conversion” *J. Catal.* **2013**, *307*, 1–17.

[4]	G. Kresse, J. Hafner, “Ab initio molecular-dynamics simulation of the liquid-metalamorphous- semiconductor transition in germanium” *Phys. Rev. B* **1994**, *49*, 14251–14269.

[5]	P. E. Blöchl, “Projector augmented-wave method” *Phys. Rev. B* **1994**, *50*, 17953–17979.

[6]	J. P. Perdew, K. Burke, M. Ernzerhof, “Generalized gradient approximation made simple” *Phys. Rev. Lett.* **1996**, *77*, 3865–3868.

[7]	M. P. Teter, M. C. Payne, D. C. Allan, “Solution of Schrödingers equation for large systems” *Phys. Rev. B* **1989**, *40*, 12255–12263.

[8]	S. Grimme, J. Antony, S. Ehrlich, H. Krieg, “A consistent and accurate ab initio parametrization of density functional dispersion correction (DFT-D) for the 94 elements H-Pu” *J. Chem. Phys.* **2010**, *132*, 154104.

[9]	S. Grimme, S. Ehrlich, L. Goerigk, “Effect of the damping function in dispersion corrected density functional theory” *J. Comput. Chem.* **2011**, *32*, 1456–1465.

[10]	B. Qiao, A. Wang, X. Yang, L. F. Allard, Z. Jiang, Y. Cui, J. Liu, J. Li, T. Zhang, “Single-atom catalysis of CO oxidation using Pt_1_/FeO_x_” *Nat. Chem.* **2011**, *3*, 634–641.

[11]	L. M. Sandratskii, M. Uhl, J. Kübler, “Band theory for electronic and magnetic properties of α-Fe2O3” *J. Phys. Condens. Matter* **1996**, *8*, 983–989.

[12]	G. Rollmann, A. Rohrbach, P. Entel, J. Hafner, “First-principles calculation of the structure and magnetic phases of hematite” *Phys. Rev. B - Condens. Matter Mater. Phys.* **2004**, *69*, 1–12.

[13]	A. Michaelides, Z. P. Liu, C. J. Zhang, A. Alavi, D. A. King, P. Hu, “Identification of general linear relationships between activation energies and enthalpy changes for dissociation reactions at surfaces” *J. Am. Chem. Soc.* **2003**, *125*, 3704–3705.

[14]	A. Alavi, P. Hu, T. Deutsch, P. L. Silvestrelli, J. Hutter, “CO oxidation on Pt(111): An Ab initio density functional theory study” *Phys. Rev. Lett.* **1998**, *80*, 3650–3653.

[15]	Z. P. Liu, P. Hu, “General rules for predicting where a catalytic reaction should occur on metal surfaces: A density functional theory study of C-H and C-O bond breaking/making on flat, stepped, and kinked metal surfaces” *J. Am. Chem. Soc.* **2003**, *125*, 1958–1967.

[16]	Excelitas Technologies, https://www.excelitas.com.

[17]	O. Elbanna, M. Zhu, M. Fujitsuka, T. Majima, “Black Phosphorus Sensitized TiO_2_ Mesocrystal Photocatalyst for Hydrogen Evolution with Visible and Near-Infrared Light Irradiation” *ACS Catal.* **2019**, *9*, 3618–3626.

[18]	W. Jiang, S. Bai, L. Wang, X. Wang, L. Yang, Y. Li, D. Liu, X. Wang, Z. Li, J. Jiang, Y. Xiong, “Integration of Multiple Plasmonic and Co-Catalyst Nanostructures on TiO_2_ Nanosheets for Visible-Near-Infrared Photocatalytic Hydrogen Evolution” *Small* **2016**, *12*, 1640–1648.

[19]	Z. Lian, Y. Kobayashi, J. J. M. Vequizo, C. S. K. Ranasinghe, A. Yamakata, T. Nagai, K. Kimoto, K. Kobayashi, K. Tanaka, T. Teranishi, M. Sakamoto, “Harnessing infrared solar energy with plasmonic energy upconversion” *Nat. Sustain.* **2022**, *5*, 1092–1099.

[20]	M. Zhu, Y. Osakada, S. Kim, M. Fujitsuka, T. Majima, “Black phosphorus: A promising two dimensional visible and near-infrared-activated photocatalyst for hydrogen evolution” *Appl. Catal. B Environ.* **2017**, *217*, 285–292.

[21]	M. Zhu, C. Zhai, M. Fujitsuka, T. Majima, “Noble metal-free near-infrared-driven photocatalyst for hydrogen production based on 2D hybrid of black Phosphorus/WS_2_” *Appl. Catal. B Environ.* **2018**, *221*, 645–651.

[22]	J. Jiang, X. Tang, S. Zhou, J. Ding, H. Zhou, F. Zhang, D. Zhang, T. Fan, “Synthesis of visible and near infrared light sensitive amorphous titania for photocatalytic hydrogen evolution” *Green Chem.* **2016**, *18*, 2056–2062.

[23]	A. Gannoruwa, K. Niroshan, O. A. Ileperuma, J. Bandara, “Infrared radiation active, novel nanocomposite photocatalyst for water splitting” *Int. J. Hydrogen Energy* **2014**, *39*, 15411–15415.

[24]	M. Zhu, S. Kim, L. Mao, M. Fujitsuka, J. Zhang, X. Wang, T. Majima, “Metal-Free Photocatalyst for H2 Evolution in Visible to Near-Infrared Region: Black Phosphorus/Graphitic Carbon Nitride” *J. Am. Chem. Soc.* **2017**, *139*, 13234–13242.

[25]	M. Zhu, X. Cai, M. Fujitsuka, J. Zhang, T. Majima, “Au/La_2_Ti_2_O_7_ Nanostructures Sensitized with Black Phosphorus for Plasmon-Enhanced Photocatalytic Hydrogen Production in Visible and Near-Infrared Light” *Angew. Chem. Int. Ed.* **2017**, *129*, 2096–2100.

[26]	Y. Zhang, L. Wang, S. H. Park, X. Kong, X. Lan, Z. Song, J. Shi, “Single near-infrared-laser driven Z-scheme photocatalytic H_2_ evolution on upconversion material@Ag3PO4@black phosphorus” *Chem. Eng. J.* **2019**, *375*, 121967.

[27]	C. Xing, Y. Liu, Y. Zhang, X. Wang, P. Guardia, L. Yao, X. Han, T. Zhang, J. Arbiol, L. Soler, Y. Chen, K. Sivula, N. Guijarro, A. Cabot, J. Llorca, “A Direct Z-Scheme for the Photocatalytic Hydrogen Production from a Water Ethanol Mixture on CoTiO_3_/TiO_2_ Heterostructures” *ACS Appl. Mater. Interfaces* **2021**, *13*, 449–457.

[28]	M. Tahir, “Ni/MMT-promoted TiO_2_ nanocatalyst for dynamic photocatalytic H_2_ and hydrocarbons production from ethanol-water mixture under UV-light” *Int. J. Hydrogen Energy* **2017**, *42*, 28309–28326.

[29]	M. J. Sampaio, J. W. L. Oliveira, C. I. L. Sombrio, D. L. Baptista, S. R. Teixeira, S. A. C. Carabineiro, C. G. Silva, J. L. Faria, “Photocatalytic performance of Au/ZnO nanocatalysts for hydrogen production from ethanol” *Appl. Catal. A Gen.* **2016**, *518*, 198–205.

[30]	S. Liu, Y. Meng, C. Luo, H. Zhu, Y. Song, H. Zhang, T. Wu, “Green hydrogen and acetaldehyde production via photo-induced thermal dehydrogenation of bioethanol over a highly dispersed CuS/TiO_2_ catalyst” *Int. J. Hydrogen Energy* **2024**, *86*, 1414–1423.

[31]	S. Luo, H. Song, D. Philo, M. Oshikiri, T. Kako, J. Ye, “Solar-driven production of hydrogen and acetaldehyde from ethanol on Ni-Cu bimetallic catalysts with solar-to-fuels conversion efficiency up to 3.8 %” *Appl. Catal. B Environ.* **2020**, *272*, 118965.

[32]	B. Xia, B. He, J. Zhang, L. Li, Y. Zhang, J. Yu, J. Ran, S. Qiao, “TiO_2_ /FePS_3_ S‐Scheme Heterojunction for Greatly Raised Photocatalytic Hydrogen Evolution” *Adv. Energy Mater.* **2022**, *12*, 2201449.

[33]	H. Lu, J. Zhao, L. Li, L. Gong, J. Zheng, L. Zhang, Z. Wang, J. Zhang, Z. Zhu, “Selective oxidation of sacrificial ethanol over TiO_2_-based photocatalysts during water splitting” *Energy Environ. Sci.* **2011**, *4*, 3384.

[34]	X. Zhang, D. Luo, Y. Liu, X. Wang, H. Hu, J. Ye, D. Wang, “Efficient photothermal alcohol dehydration over a plasmonic W_18_O_49_ nanostructure under visible-to-near-infrared irradiation” *J. Photochem. Photobiol. A Chem.* **2023**, *441*, 114728.

[35]	A. V. Puga, A. Forneli, H. García, A. Corma, “Production of H_2_ by Ethanol Photoreforming on Au/TiO_2_” *Adv. Funct. Mater.* **2014**, *24*, 241–248.

[36]	H. Zhang, H. R. Tan, S. Jaenicke, G. K. Chuah, “Highly efficient and robust Cu catalyst for non-oxidative dehydrogenation of ethanol to acetaldehyde and hydrogen” *J. Catal.* **2020**, *389*, 19–28.

[37]	J. Shan, N. Janvelyan, H. Li, J. Liu, T. M. Egle, J. Ye, M. M. Biener, J. Biener, C. M. Friend, M. Flytzani-Stephanopoulos, “Selective non-oxidative dehydrogenation of ethanol to acetaldehyde and hydrogen on highly dilute NiCu alloys” *Appl. Catal. B Environ.* **2017**, *205*, 541–550.

[38]	S. Tayrabekova, P. Mäki-Arvela, M. Peurla, P. Paturi, K. Eränen, G. E. Ergazieva, A. Aho, D. Y. Murzin, K. Dossumov, “Catalytic dehydrogenation of ethanol into acetaldehyde and isobutanol using mono- and multicomponent copper catalysts” *Comptes Rendus. Chim.* **2017**, *21*, 194–209.

[39]	I. C. Freitas, S. Damyanova, D. C. Oliveira, C. M. P. Marques, J. M. C. Bueno, “Effect of Cu content on the surface and catalytic properties of Cu/ZrO_2_ catalyst for ethanol dehydrogenation” *J. Mol. Catal. A Chem.* **2014**, *381*, 26–37.

[40]	J. Pang, M. Zheng, C. Wang, X. Yang, H. Liu, X. Liu, J. Sun, Y. Wang, T. Zhang, “Hierarchical Echinus-like Cu-MFI Catalysts for Ethanol Dehydrogenation” *ACS Catal.* **2020**, *10*, 13624–13629.

[41]	M. Śliwa, J. Podobiński, D. Rutkowska-Zbik, J. Datka, “In situ IR studies on ethanol transformations over CuO, CuO/ZrO_2_ and CuO/ZrO_2_/ZnO catalysts modified with NiO” *J. Mol. Struct.* **2022**, *1257*, 132581.

[42]	P. J. Soininen, K.-E. Elers, V. Saanila, S. Kaipio, T. Sajavaara, S. Haukka, “Reduction of Copper Oxide Film to Elemental Copper” *J. Electrochem. Soc.* **2005**, *152*, G122.

[43]	H. Yousefzadeh, S. E. Bozbag, V. Sushkevich, J. A. Van Bokhoven, “Stepwise conversion of methane to methanol over Cu-mordenite prepared by supercritical and aqueous ion exchange routes and quantification of active Cu species by H_2_ -TPR” *Catal. Commun.* **2023**, *174*, 106574.

[44]	M. Luo, P. Fang, M. He, Y. Xie, “In situ XRD, Raman, and TPR studies of CuO/Al_2_O_3_ catalysts for CO oxidation” *J. Mol. Catal. A Chem.* **2005**, *239*, 243–248.

[45]	Q. Liu, M. Wen, Y. Guo, S. Song, G. Li, T. An, “Efficient Catalytic Combustion of Cyclohexane over PdAg/Fe_2_O_3_ Catalysts under Low-Temperature Conditions: Establishing the Degradation Mechanism Using PTR-TOF-MS and in Situ DRIFTS” *ACS Appl. Mater. Interfaces* **2022**, *14*, 55503–55516.

[46]	S. Sarina, H.-Y. Zhu, Q. Xiao, E. Jaatinen, J. Jia, Y. Huang, Z. Zheng, H. Wu, “Viable Photocatalysts under Solar-Spectrum Irradiation: Nonplasmonic Metal Nanoparticles” *Angew. Chemie Int. Ed.* **2014**, *53*, 2935–2940.

[47]	L. Zhou, J. M. P. Martirez, J. Finzel, C. Zhang, D. F. Swearer, S. Tian, H. Robatjazi, M. Lou, L. Dong, L. Henderson, P. Christopher, E. A. Carter, P. Nordlander, N. J. Halas, “Light-driven methane dry reforming with single atomic site antenna-reactor plasmonic photocatalysts” *Nat. Energy* **2020**, *5*, 61–70.

[48]	Q. Darugar, W. Qian, M. A. El-Sayed, M.-P. Pileni, “Size-Dependent Ultrafast Electronic Energy Relaxation and Enhanced Fluorescence of Copper Nanoparticles” *J. Phys. Chem. B* **2006**, *110*, 143–149.

[49]	M. Holtz, T. Prokofyeva, M. Seon, K. Copeland, J. Vanbuskirk, S. Williams, S. A. Nikishin, V. Tretyakov, H. Temkin, “Composition dependence of the optical phonon energies in hexagonal AlxGa1-xN” *J. Appl. Phys.* **2001**, *89*, 7977–7982.

[50]	X. Yan, C. Liu, C. A. Gadre, S. Dai, L. Gu, K. Yu, T. Aoki, R. Wu, X. Pan, “Unexpected Strong Thermally Induced Phonon Energy Shift for Mapping Local Temperature” *Nano Lett.* **2019**, *19*, 7494–7502.

[51]	A. K. Viswanath, J. I. Lee, “Exciton-phonon interactions, exciton binding energy, and their importance in the realization of room-temperature semiconductor lasers based on GaN” *Phys. Rev. B - Condens. Matter Mater. Phys.* **1998**, *58*, 16333–16339.

[52]	S. Kilina, D. Kilin, S. Tretiak, “Light-Driven and Phonon-Assisted Dynamics in Organic and Semiconductor Nanostructures” *Chem. Rev.* **2015**, *115*, 5929–5978.

[53]	A. A. Jahagirdar, N. Dhananjaya, D. L. Monika, C. R. Kesavulu, H. Nagabhushana, S. C. Sharma, B. M. Nagabhushana, C. Shivakumara, J. L. Rao, R. P. S. Chakradhar, “Structural, EPR, optical and magnetic properties of α-Fe_2_O_3_ nanoparticles” *Spectrochim. Acta - Part A Mol. Biomol. Spectrosc.* **2013**, *104*, 512–518.

[54]	J. L. Shelton, K. E. Knowles, “Polaronic optical transitions in hematite (α-Fe_2_O_3_) revealed by first-principles electron-phonon coupling” *J. Chem. Phys.* **2022**, *157*, 174703.

[55]	P. Kubelík, E. M. Zanozina, A. Pastorek, M. Ferus, L. Juha, V. E. Chernov, A. V. Naskidashvili, S. Civiš, “Argon FTIR spectra between 800 and 2000 cm^−1^: h- and i-levels and transition probabilities” *J. Quant. Spectrosc. Radiat. Transf.* **2016**, *182*, 337–345.
